# Structure–Function Insights into Quinuclidine-3-One BisQACs: Synthesis, Modulation of Bacterial Resistance, Structure–Activity Relationship, and Biological Profiling

**DOI:** 10.3390/ph18091286

**Published:** 2025-08-28

**Authors:** Antonio Sabljić, Doris Čarija, Alma Ramić, Matilda Šprung, Renata Odžak

**Affiliations:** 1 Department of Chemistry, Faculty of Science, University of Split, R. Bošković 33, 21000 Split, Croatia; antonio.sabljic@pmfst.hr (A.S.); dcrncevic@pmfst.hr (D.Č.); msprung@pmfst.hr (M.Š.); 2 Doctoral Study of Biophysics, Faculty of Science, University of Split, R. Bošković 33, 21000 Split, Croatia; 3 Department of Chemistry, Faculty of Science, University of Zagreb, Horvatovac 102a, 10000 Zagreb, Croatia; aramic@chem.pmf.hr

**Keywords:** bisquaternary ammonium compounds (bisQACs), antimicrobial resistance, membrane permeabilization, biofilm inhibition, molecular docking, cytotoxicity

## Abstract

**Background**: The increasing prevalence of antibiotic-resistant bacterial strains highlights the urgent need for new membrane-targeting antimicrobial agents. Bisquaternary ammonium compounds (bisQACs) have attracted attention for their ability to disrupt bacterial membranes more effectively than monoquaternary analogs. Quinuclidine, known for its health-beneficial properties, has previously been explored for monoQAC derivatization, but studies using natural scaffolds to generate bisQACs remain limited. **Methods**: Here, we synthesized twelve novel quinuclidine-based bisQACs, systematically varying alkyl chain and linker lengths to investigate structure–activity relationships. **Results:** Several compounds, including 2(QC_16_)_3_, 2(QC_16_)_4_, 2(QC_14_)_6_, and 2(QC_16_)_6_, exhibited strong activity against *Staphylococcus aureus* (including MRSA), *Listeria monocytogenes*, and *Escherichia coli*, with 2(QC_16_)_6_ being the most potent (MICs 5–38 µM). While cytotoxicity was observed on human RPE1 and HEK293 cells, selectivity indices indicated a favorable therapeutic window relative to reference QACs. **Conclusions:** These compounds also inhibited biofilm formation and induced rapid bacterial killing through a membrane-disruptive mode of action. Molecular docking showed that alkyl chain and linker variations modulate binding to the QacR efflux regulator, revealing a lower potential for efflux-mediated resistance. Overall, quinuclidine-based bisQACs represent promising leads for potent, selectively active next-generation antimicrobials with a reduced likelihood of resistance development.

## 1. Introduction

Quaternary ammonium compounds (QACs) are a crucial class of antiseptics and disinfectants, accounting for ~40% of the global market as of 2022 [1]. Known for their broad-spectrum antimicrobial activity, QACs are especially effective against enveloped viruses such as SARS-CoV-2 [2]. Their primary mechanism involves electrostatic interactions between the positively charged quaternary nitrogen atom and negatively charged phospholipid head groups in bacterial membranes, resulting in membrane disruption and leakage of intracellular contents [3]. This mode of action is particularly potent against Gram-positive bacteria, which lack an outer membrane barrier [4]. In contrast, Gram-negative bacteria present a greater challenge due to their outer membrane, which acts as a selective permeability barrier and limits QAC penetration [4]. As a result, higher concentrations of monoQACs like benzalkonium chloride (BAC) and cetylpyridinium chloride (CPC) are typically required to achieve similar efficacy [5]. Despite this, monoQACs remain widely used in healthcare and industry [5].

Recent studies have also emphasized the increasing ecological and health concerns associated with widespread QAC use. Their persistence in wastewater and sludge can disrupt microbial community dynamics, interfere with wastewater treatment performance, and contribute to environmental toxicity [6]. Moreover, subinhibitory exposure has been shown to promote the development of cross-resistance to clinically relevant antibiotics in both Gram-positive and Gram-negative bacteria [7,8]. These issues have fueled regulatory attention and encouraged the development of next-generation QACs with improved safety and biodegradability and reduced ecological footprint [9].

To address the limitations of monoQACs, considerable research has focused on developing more potent derivatives, notably bis- and trisQACs [10]. These molecules feature multiple quaternary ammonium centers, enhancing membrane interactions through multivalency [8]. Structure–activity relationship (SAR) studies have highlighted the critical role of dual positive charges in bisQACs, which increase electrostatic interactions with bacterial membranes compared to monoQACs [11]. BisQACs have shown improved activity against both Gram-positive and Gram-negative strains, including multidrug-resistant organisms such as methicillin-resistant *Staphylococcus aureus* (MRSA) [11].

**Figure 1:**
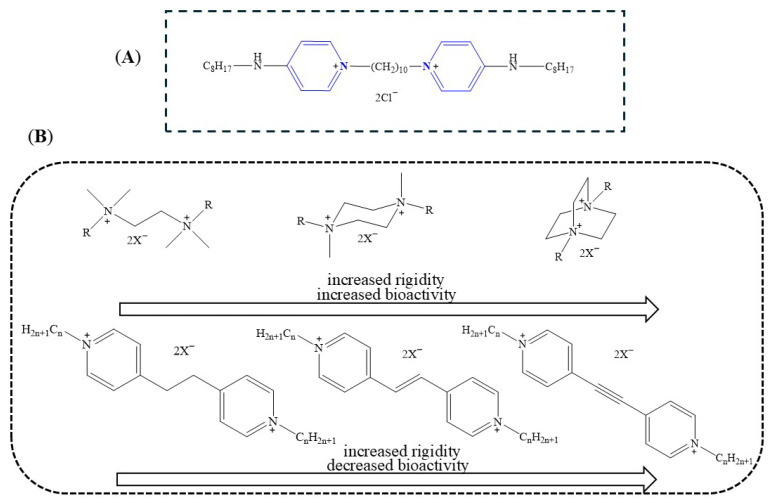
Chemical structures of the commercially available bisQAC OCT (**A**) [32] and synthesized bisQACs (**B**) [20,21], including TMED-, piperazine-, and DABCO-based scaffolds, as well as pyridinium derivatives with variable linker rigidity (alkane, alkene, alkyne).

Among the most potent antiseptics in this class is octenidine dihydrochloride (OCT), a bis-pyridinium compound introduced in the 1980s and currently marketed in formulations such as Octenisept^®^ [12]. OCT features a dimeric structure with two pyridinium rings linked via an aliphatic chain and para-positioned alkylamine groups (see Figure 1A). It displays strong antimicrobial activity against a wide range of clinically relevant pathogens, including *Staphylococcus aureus*, *Staphylococcus epidermidis*, *Proteus mirabilis*, *Klebsiella pneumoniae*, *Escherichia coli*, and *Pseudomonas aeruginosa* [13]. The long alkyl linker and dual cationic sites facilitate robust binding to bacterial membranes [14].

Subsequent advances have aimed to optimize bisQACs through structural modifications, primarily due to several limitations associated with their earlier forms. Although bisQACs exhibit potent antimicrobial activity, their practical application has been constrained by significant cytotoxicity toward mammalian cells, limited selectivity, and poor aqueous solubility caused by the presence of long hydrophobic alkyl chains [15]. Additionally, concerns regarding the potential development of microbial resistance, environmental persistence, and limited efficacy against biofilms have prompted further structural refinement [16]. Therefore, recent research has focused on balancing antimicrobial potency with improved biocompatibility, enhanced physicochemical properties, and reduced ecological impact through rational design and structural diversification [9,11]. Early studies explored variations in linker types (ether, thioether, amide, ester), revealing that most bisQACs outperformed monoQACs in antimicrobial assays [17]. These structural differences influenced membrane interaction, antimicrobial potency, and biodegradability. For instance, ether and thioether linkers enhanced lipophilicity and conformational freedom, while amide and ester linkers introduced polarity and hydrolytically labile sites, enabling fine-tuning of bioactivity and biocompatibility [18,19].

BisQACs with more rigid cores, particularly those based on DABCO, exhibit significantly lower MIC values than analogs with flexible scaffolds such as TMEDA or moderately rigid piperazine (see Figure 1B) [20]. This is attributed to enhanced preorganization of the charged centers, facilitating effective membrane interactions. Excessive linker length or flexibility reduces bioactivity by increasing the spatial distance between quaternary ammonium groups. Optimal antimicrobial effects were observed when charged centers remained in close proximity, supporting efficient membrane disruption (see Figure 1B) [21]. Optimal antimicrobial activity of bisQAC antiseptics strongly depends on both linker length and rigidity, as well as the spatial proximity of the positively charged centers. Shorter linkers that position the cationic groups closer together enhance cooperative binding to the negatively charged bacterial membrane, increasing membrane disruption and bactericidal efficacy [22]. This is likely due to the better spatial arrangement and flexibility of positive charges, which facilitates stronger membrane interactions. Overall, linker flexibility and optimal charge positioning are key parameters in designing potent and broad-spectrum bisQAC antiseptics [21]. More recent studies demonstrated that amide-containing pyridinium salts, which increase cationic charge density, enhance microbial membrane adsorption [23]. Subsequent work showed that incorporating mixed alkyl-aromatic linkers can further improve antifungal activity [24].

One of the key bacterial resistance mechanisms to quaternary ammonium compounds (QACs) involves the QacR transcriptional repressor, which regulates the expression of the multidrug efflux pump gene *qacA* in *Staphylococcus aureus* [7,25]. QacR binds to structurally diverse QACs and induces conformational changes that relieve repression of *qacA*, leading to active efflux of toxic compounds [26]. While monoQACs have been shown to interact with QacR and induce resistance-related gene expression, data regarding bisQACs and their interaction with QacR remain limited. Existing studies suggest that bisQACs, due to their bulkier and more rigid structures, may bind differently to QacR or even evade recognition altogether, potentially reducing the likelihood of efflux-mediated resistance. Although no direct evidence for bisQAC–QacR interactions is available, structural analyses with other bulky bivalent cations (e.g., pentamidine, hexamidine) have shown that the QacR binding pocket can undergo conformational rearrangements to accommodate larger ligands, supporting the possibility of an altered recognition mode [27]. However, resistance testing results have been variable, and further insights are needed to clarify the relationship between bisQAC structure and QacR-mediated regulation. This gap highlights the importance of molecular docking studies to evaluate the binding modes of newly synthesized bisQACs to QacR and assess their potential to induce resistance.

In addition to QacR-mediated efflux in Gram-positive bacteria, Gram-negative pathogens present an even greater challenge due to their intrinsic resistance mechanisms, including the presence of an outer membrane barrier, low membrane permeability, and efficient efflux systems such as AcrAB-TolC [7,8]. These factors limit the intracellular accumulation of QACs and reduce their antimicrobial efficacy. Despite their potency, the broader application of bisQACs has been hindered by issues such as cytotoxicity, poor water solubility, and limited activity against Gram-negative species and biofilms [28]. Consequently, recent efforts have focused on rational structural modifications—particularly the design of different linker types, variations in molecular flexibility, and adjustments in charge distribution—to enhance membrane interactions, improve selectivity, and overcome resistance mechanisms. These modifications aim to optimize the physicochemical properties of bisQACs while minimizing their susceptibility to known bacterial defense systems [29,30,31].

In this study, we present the design, synthesis, and comprehensive biological evaluation of a new series of bisquaternary ammonium compounds (bisQACs) featuring a quinuclidine core. These compounds exhibit potent antibacterial and antibiofilm activities, favorable cytotoxicity profiles on human cell lines, and reduced susceptibility to efflux-mediated resistance mechanisms. Mechanistic studies confirmed their membrane-disruptive mode of action, while molecular docking analyses demonstrated that systematic variations in alkyl chain length and linker dimensions significantly modulate binding affinity to the QacR transcriptional repressor—a key regulator implicated in the activation of multidrug efflux systems in pathogenic bacteria.

Furthermore, we employed flow cytometry to gain deeper insight into the compounds’ effects on bacterial membrane integrity and cell viability. This technique enabled precise, quantitative assessment of membrane depolarization and permeability, supporting our findings from conventional biochemical assays and confirming the proposed mechanism of action.

## 2. Results and Discussion

### 2.1. Synthesis of BisQAC

**Scheme 1:**
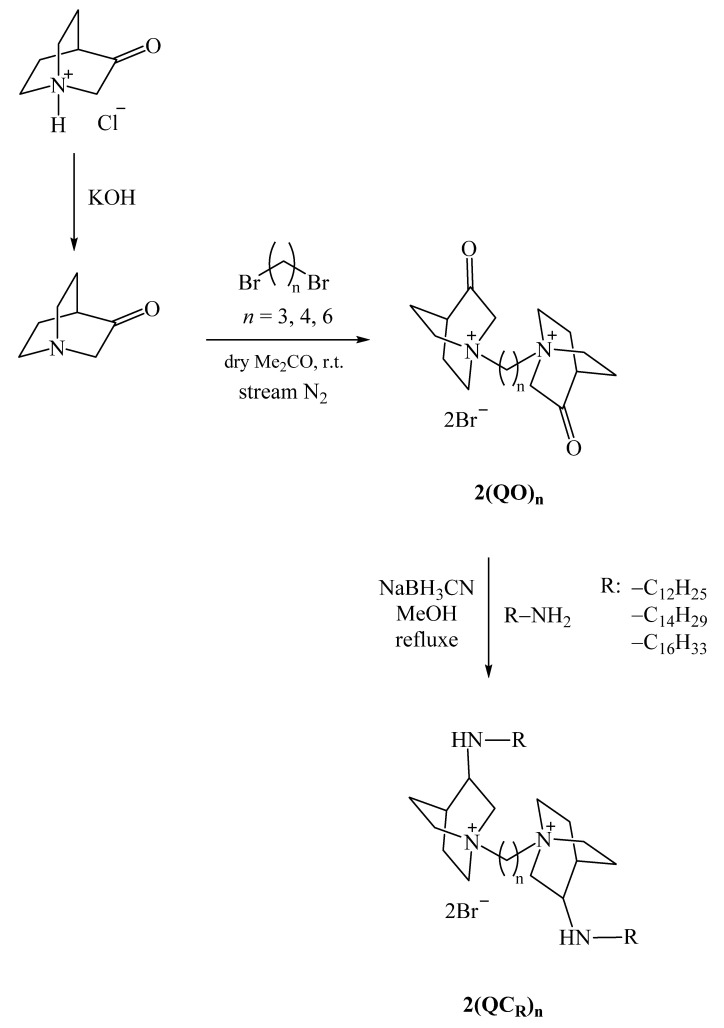
Synthetic pathways of newly synthesized bisQACs derived from quinuclidine-3-one.

As part of the ongoing progression of our research, we synthesized a series of bisQACs derived from quinuclidine-3-one. These compounds were synthesized with varying linker lengths of three, four, and six carbon atoms through the Menshutkin reaction, a well-established method for the formation of quaternary ammonium salts [33]. The selection of these linker lengths was intended to systematically investigate the impact of chain length on the antimicrobial activity of the resulting bisQACs. Following the successful synthesis of the bisQACs, we further modified the compounds via a reductive amination reaction to introduce alkyl chains of varying lengths at the C-3 carbon position at quinuclidine (see Scheme 1). The alkylation process was carried out with different alkyl chain (C_12_, C_14_, and C_16_) precursors to explore the effect of alkyl chain length on the overall bioactivity and membrane-interacting properties of the bisQACs. This dual-modification approach allowed for the systematic variation of both the linker length and the alkyl chain length, providing a robust set of compounds for subsequent biological evaluation.

These bisQAC derivatives, incorporating variations in linker length, molecular flexibility, and alkyl chain structure, represent a new class of compounds subjected to comprehensive biological profiling. Their antimicrobial activity will be evaluated against a broad spectrum of Gram-positive and Gram-negative bacterial strains, alongside toxicity assessments to identify promising candidates for further development. In this study, molecular flexibility refers to the rotational freedom of the linker connecting the two quaternary ammonium centers in bisQAC compounds. We hypothesized that linker flexibility influences antimicrobial activity by modulating membrane interactions and evasion of resistance mechanisms. Flexibility was assessed through molecular modeling and structure–activity relationship analysis. The data generated will provide key insights into how molecular flexibility, spacer length, and alkyl chain variation influence both the antimicrobial potency of bisQACs and their potential to induce or bypass bacterial resistance pathways, particularly those mediated by efflux regulation through the QacR repressor system.

### 2.2. Antibacterial Evaluation (MICs)

The antibacterial activity of bisquaternary ammonium compounds (bisQACs) derived from quinuclidin-3-one scaffold was assessed by determining the minimum inhibitory concentrations (MICs) against a panel of clinically relevant bacterial strains. This panel included six Gram-positive species—*Staphylococcus aureus* (ATCC 25923, methicillin-resistant ATCC 33591), *Bacillus cereus* (ATCC 14579), *Listeria monocytogenes* (ATCC 7644), and *Enterococcus faecalis* (ATCC 29212)—and three Gram-negative species—*Escherichia coli* (ATCC 25922), *Salmonella enterica* (food isolate), and *Pseudomonas aeruginosa* (ATCC 27853). Initial precursors of quinuclidin-3-one-based bisQACs with short alkyl linkers (three, four, and six carbon atoms) were biologically inactive (MIC > 75 µM), likely due to their overall polarity and rigid spatial arrangement of quaternary centers, which impaired membrane permeation (Table 1). However, since these non-alkylated precursors differ substantially from fully alkylated analogs in both polarity and lipophilicity, linker length alone cannot be directly correlated with bioactivity in this series. To assess the true impact of spacer length, comparisons must be made within structurally consistent series—particularly among alkylated bisQACs with identical alkyl side chains but varying linker lengths. In contrast, novel derivatives bearing various alkyl chains at the C-3 position of the quinuclidine ring exhibited significant antibacterial activity against both Gram-positive and Gram-negative bacterial strains. Structure–activity relationship (SAR) analysis revealed that the spatial distance and flexibility between the two quaternary nitrogen centers (i.e., the conformational freedom of the linker, ability to rotate around σ-bonds) strongly modulate antibacterial potency [20]. Short linkers may induce molecular rigidity, reducing adaptability for effective membrane interaction, whereas excessively long linkers could result in loss of structural coherence [34]. An optimal linker length was found to favor enhanced binding to bacterial membranes, increased membrane permeabilization, and a consequent bactericidal effect [35]. Variation in the length of the hydrophobic alkyl chains further influenced antibacterial efficacy. Compounds with longer aliphatic chains generally showed improved activity, indicating a positive correlation between alkyl chain length and membrane disruption capacity. Active bisQACs exhibited MIC values ranging from 5 to 38 μM against Gram-positive bacteria (*S. aureus* ATCC 25923 and ATCC 33591, *B. cereus*, *L. monocytogenes*, and *E. faecalis*) and from 19 to 38 μM against *E. coli*. When compared with the commercial quaternary ammonium compound BAB (benzyldodecyldimethylammonium bromide), 2(QC_16_)_6_ displayed enhanced activity against *Staphylococcus aureus* ATCC 33591 (MIC values of 5 µM for 2(QC_16_)_6_ and 25 µM for BAB) as well as *Escherichia coli* ATCC 25922 (19 µM and 63 µM, respectively). Similar activity was observed against *S. aureus* ATCC 25923 (9 µM and 10 µM). For the *Bacillus cereus*, *Listeria monocytogenes*, and clinical *MRSA* isolate, MIC values of 19, 38, and 38 µM were obtained for 2(QC_16_)_6_, compared with 10, 25, and 25 µM for BAB. In the case of *Enterococcus faecalis*, both compounds showed comparable efficacy (15 µM and 19 µM). Against *Salmonella enterica*, BAB inhibited growth at 50 µM, while 2(QC_16_)_6_ required 75 µM. Both agents were largely inactive against *Pseudomonas aeruginosa*. Collectively, these findings identify 2(QC_16_)_6_ as the most active derivative in the series, with clear advantages over BAB in selected Gram-positive and Gram-negative strains. Notably, certain bisQACs exhibited stronger activity against methicillin-resistant *S. aureus* (MRSA, ATCC 33591) compared to the methicillin-susceptible ATCC 25923 strain. This paradoxical finding may be explained by adaptive changes in membrane architecture associated with resistance phenotypes, including altered membrane composition, increased surface negativity, and the presence of active efflux systems. These adaptations may enhance electrostatic interactions with cationic bisQACs, potentially facilitating their membrane insertion and lysis [36]. Furthermore, MRSA strains often display a more rigid membrane structure as a defense mechanism, which paradoxically may increase susceptibility to membrane-disruptive agents like bisQACs [36]. Conversely, the membrane of susceptible strains tends to be more fluid and less negatively charged, which may reduce the initial affinity for bisQACs but allows more efficient membrane repair mechanisms, ultimately promoting bacterial survival [37]. These observations underscore the potential of quinuclidine-based bisQACs as promising antimicrobials against resistant pathogens, particularly those with altered membrane physicochemical properties resulting from selective pressure imposed by antibiotic use.

### 2.3. Time-Resolved Analysis of Bacterial Growth Kinetics

The presence of an antimicrobial agent can significantly alter bacterial growth dynamics, offering insights into the onset, duration, and intensity of action. While standard assays such as broth microdilution provide MIC values, they lack temporal resolution. In contrast, kinetic growth analysis enables continuous monitoring of bacterial responses, offering a more detailed view of pharmacodynamic behavior [38].

This approach captures key parameters such as lag phase duration, exponential growth rate, and possible adaptive responses, including tolerance or early resistance. The lag phase, a metabolically active period preceding visible proliferation, reflects bacterial adaptation to environmental and antimicrobial stress [39].

By tracking these dynamics, kinetic profiling reveals sub-MIC effects and mechanistic insights that complement conventional susceptibility testing. To evaluate the performance of selected bisquaternary ammonium compounds (bisQACs), growth inhibition kinetics were assessed in *Staphylococcus aureus* ATCC 25923 and *Listeria monocytogenes* ATCC 7644. These findings underscore the impact of molecular structure on bacterial growth kinetics, particularly in terms of growth delay and post-treatment effects.

**Figure 2:**
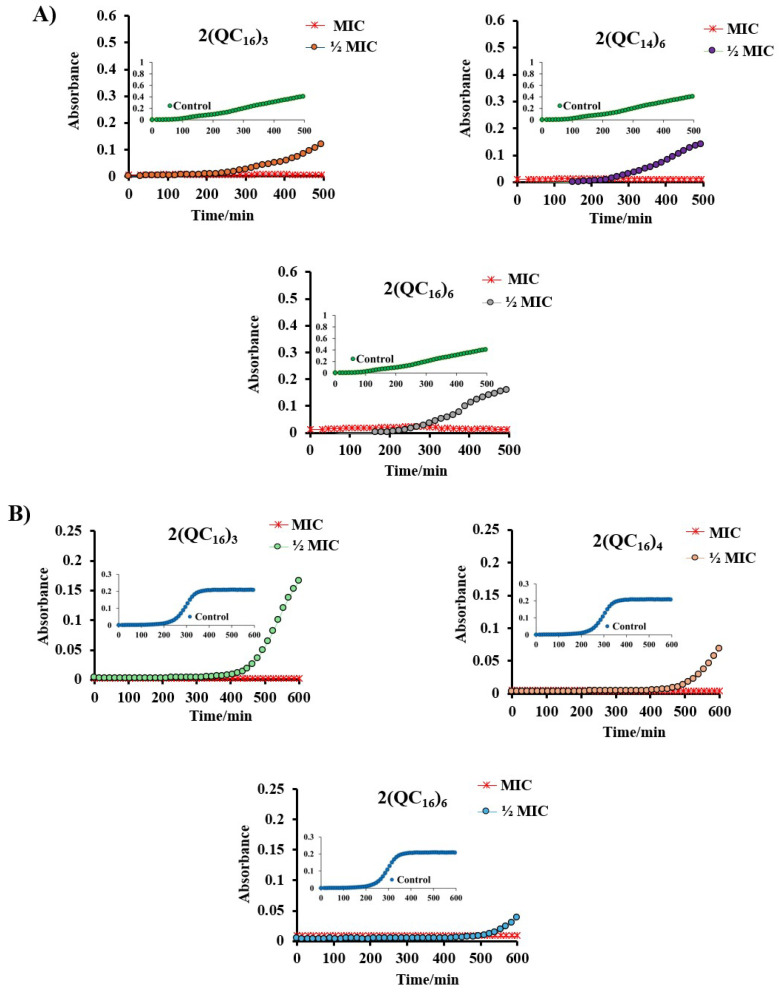
Time-resolved growth kinetics of *Staphylococcus aureus* (**A**) and *Listeria monocytogenes* (**B**) were monitored over a defined incubation period in the absence of antibacterial agents (control) and in the presence of the most potent bisQACs at MIC and ½ MIC concentrations.

At the minimum inhibitory concentration (MIC), the tested bisquaternary ammonium compounds (bisQACs) fully suppressed the growth of *Staphylococcus aureus* ATCC 25923 throughout the monitored 500 min interval (Figure 2A). Growth kinetics at subinhibitory concentrations revealed comparable lag phases across all compounds; however, differences emerged during the exponential phase. Specifically, compound 2(QC_14_)_6_ exhibited a shallower growth curve slope relative to its structural analogs, 2(QC_16_)_3_ and 2(QC_16_)_6_, indicating a reduced bacterial proliferation rate. This observation suggests possible variations in the compound’s mechanism of action or in its interaction with the bacterial membrane, potentially arising from differences in hydrophobicity, charge distribution, or membrane penetration efficiency. The untreated control exhibited characteristic growth kinetics of *S. aureus*, comprising an initial lag phase, a subsequent exponential proliferation phase, and a final plateau corresponding to the stationary phase. Further kinetic and structural analyses are required to elucidate these distinctions and their implications for antimicrobial activity.

The bisquaternary ammonium compounds 2(QC_16_)_3_, 2(QC_16_)_4_, and 2(QC_16_)_6_ exhibited a pronounced inhibitory effect on the growth of *Listeria monocytogenes* (Figure 2B). Analysis of subinhibitory concentrations revealed that the lag phase was most prolonged in the presence of the compound with a six-carbon linker, followed by the four-carbon linker, while the shortest delay was observed with the three-carbon linker. Consistent with previous findings reported by other authors, these results suggest that increased spatial separation between the quaternary nitrogen centers within the molecule contributes to enhanced and prolonged antibacterial activity. The untreated control displayed typical growth dynamics of *L. monocytogenes*, characterized by an initial lag phase, followed by exponential growth, and a plateau as the culture reached the stationary phase.

### 2.4. Potential for the Development of Bacterial Resistance

In recent years, rising resistance to quaternary ammonium compounds (QACs) has become a major public health issue. Among the identified mechanisms, efflux-pump-mediated resistance plays a key role. These membrane-associated transport proteins export antimicrobial agents from bacterial cells, reducing intracellular drug concentrations. Since the 1990s, multiple efflux systems have been characterized in Gram-positive bacteria, including MRSA. Unlike many resistance determinants, efflux pumps are often intrinsic and encoded by operons regulated by transcriptional repressors [40,41].

The most common QAC resistance genes in Gram-positive bacteria are qacA and qacB, which encode MFS transporters QacA and QacB. Their expression is repressed by QacR, a transcriptional regulator that modulates efflux pump expression in multidrug-resistant strains [42,43].

This study aimed to evaluate the impact of the efflux pump inhibitor CCCP on the antimicrobial activity of potent bisquaternary ammonium compounds against *Staphylococcus aureus* ATCC 33591 (MRSA), a strain carrying qac genes.

The tested bisquaternary ammonium compound (bisQAC) candidates exhibited identical minimum inhibitory concentration (MIC) values, suggesting exceptional stability in the presence of the bacterium’s intrinsic resistance mechanisms. This observation implies that bisQACs act as poor substrates for Qac efflux pumps, thereby significantly reducing the likelihood of resistance development via this mechanism. The structural features of bisQACs may account for their reduced susceptibility to efflux-mediated extrusion. BisQACs possess two quaternary nitrogen centers, resulting in an overall increase in molecular positive charge. This heightened cationic nature hinders recognition by efflux pumps, which are typically optimized for smaller and less charged molecules.

In addition to structural factors, the decreased vulnerability of bisQACs to efflux may also stem from their mode of action. QACs are known to exert antimicrobial activity primarily by disrupting bacterial membranes through interactions with negatively charged lipid components such as phospholipids and teichoic acids in the cell wall [44]. The increased positive charge of bisQACs may enhance membrane binding and permeabilization, leading to more rapid and effective bactericidal action. Rapid membrane disruption minimizes compound exposure to efflux systems, further reducing the potential for active expulsion from the cell [35,45].

**Figure 3:**
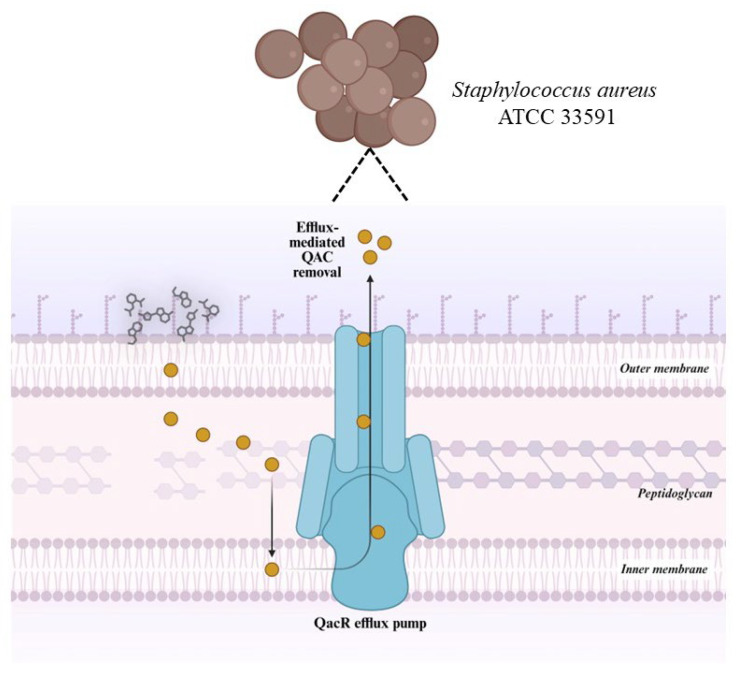
Mechanism of efflux-mediated resistance in *Staphylococcus aureus* ATCC 33591 via the QacR-regulated efflux pump. This figure was created using BioRender (www.biorender.com, accessed on 26 June 2025).

As illustrated in Figure 3, conventional QACs are actively removed by QacR-regulated efflux pumps, which decreases intracellular drug levels. In contrast, bisQACs maintain activity by avoiding efflux-mediated removal and exert enhanced membrane disruption due to their two positive charges. This dual advantage—reduced efflux susceptibility and stronger membrane-targeting activity—underscores their potential as next-generation antimicrobials with minimal risk of resistance development.

Experimental results indicate that bisQACs do not display significant shifts in MIC values in the presence of efflux pump inhibitors, providing additional evidence of their resistance to efflux-based mechanisms. This property makes them especially promising candidates for antimicrobial therapy, as they lower the risk of selecting for resistant bacterial populations.

Moreover, these findings align with prior studies suggesting that polyQACs, due to their polycationic nature and structural rigidity, are not subject to the same resistance pathways as monoQACs [35]. Wuest et al. have shown that multiple quaternization sites can limit compound interaction with the QacR transcriptional repressor, thereby reducing the induction of efflux gene expression. As a result, bisQACs not only persist intracellularly but also reduce the risk of promoting resistance development [46]. In conclusion, the data indicate that bisQACs are among the least likely candidates to drive the emergence of bacterial resistance, owing to their low affinity for efflux recognition and their distinct membrane-targeting mode of action. These insights form a foundation for future efforts to optimize bisQAC structures as next-generation antimicrobial agents with minimal risk of resistance development.

**Figure 4:**
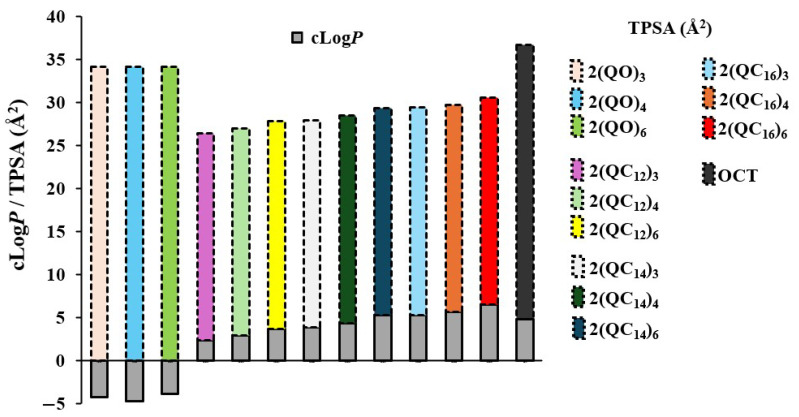
Graphical representation of lipophilicity (cLog*P*) and topological polar surface area (TPSA/Å2) values for a series of bisQACs derivatives of quinuclidine. TPSA is shown as the colored portion of each bar, while cLog*P* corresponds to the lower grey segment.

The bisquaternary ammonium derivatives exhibited elevated consensus log*P* (cLog*P*) values, indicative of their pronounced hydrophobic character. Increased lipophilicity enhances the compounds tendency to partition into lipid phases, including biological membranes, thereby facilitating membrane permeability [47]. Molecules with higher cLog*P* values typically demonstrate improved bioavailability in systems where membrane interaction is critical to pharmacological activity [48]. Furthermore, the hydrophobic nature of these compounds may promote interactions with non-polar domains of proteins, enzymes, or receptors, which can significantly influence their biological profile and pharmacodynamic potential [49]. Their affinity for lipid environments suggests a heightened capacity for engaging with membrane components, a feature that may be advantageous in therapeutic applications requiring efficient translocation across biological barriers, as shown in Figure 4.

### 2.5. Minimal Biofilm Inhibition Concentrations (MBICs)

Bacterial biofilms represent highly organized microbial communities embedded in a self-produced protective extracellular matrix, which confers enhanced resistance to antimicrobial agents and evasion of host immune defenses. Biofilms can colonize both biotic and abiotic surfaces, including medical devices, dental surfaces, and chronic wounds, and are a major contributor to persistent infections and therapeutic failure. Therefore, the development of antibacterial agents that are effective not only against planktonic bacterial cells but also against mature biofilms is crucial to improving treatment outcomes and limiting the emergence of antimicrobial resistance. The ability to form biofilms is a key factor in the persistence of bacterial pathogens under both clinical and environmental conditions, contributing to the chronicity of infections and reduced efficacy of conventional antimicrobial therapies. A comprehensive understanding of biofilm formation dynamics, structural organization, and resistance mechanisms is essential for the development of innovative strategies aimed at eradicating and preventing biofilm-associated infections [50].

**Figure 5:**
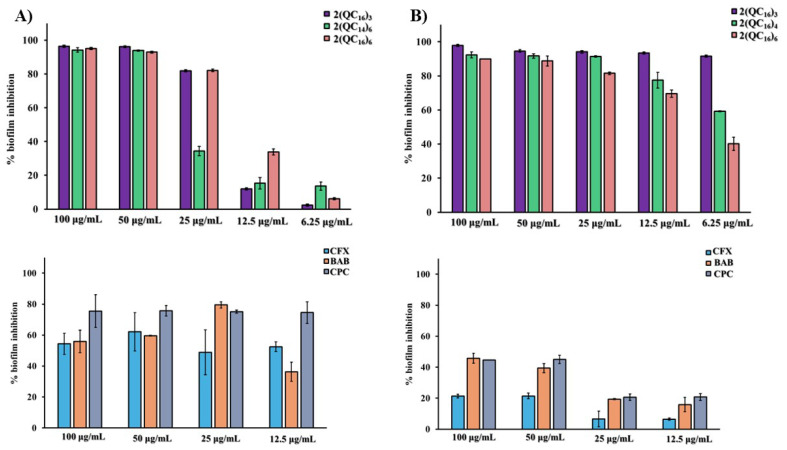
Biofilm inhibition of bacterial strains (**A**) *Staphylococcus aureus* ATCC 25923 and (**B**) *Listeria monocytogenes* ATCC 7644 following treatments with bisQAcs of quinuclidine−3−one and commercial antimicrobial agents. The percentage of biofilm inhibition was quantified at various concentrations of the tested compounds and compared to the untreated control group. Results are presented as mean ± standard deviation, calculated from at least three independent experiments, each performed in triplicate.

In this study, the minimal biofilm inhibition concentrations (MBICs) of synthesized bisquaternary ammonium compounds (bisQACs) were evaluated at five different concentrations (6.25, 12.5, 25, 50, and 100 µg/mL), Figure 5. The results were expressed as the percentage of inhibited bacterial biofilm formation and compared with the activity of two commercial quaternary ammonium compounds—benzyldimethyldodecylammonium bromide (BAB) and cetylpyridinium chloride (CPC)—as well as with the reference *β*-lactam antibiotic cefotaxime (CFX). The aim of the study was to assess the potential of bisQACs in reducing biofilm formation and enhancing antimicrobial efficacy, with a particular focus on their application in combatting persistent and multidrug-resistant bacterial infections.

This study provides critical insights into the potential of cationic amphiphilic quaternary ammonium compounds (QACs) as biofilm inhibition agents against *Staphylococcus aureus* and *Listeria monocytogenes*, which may contribute to improved treatment and prevention strategies for biofilm-associated persistent infections. Given the therapeutic challenges posed by biofilm-related infections, particularly in immunocompromised patients, the development of novel agents targeting biofilm disruption could have significant clinical implications [51]. Further investigation into the mechanisms of action of these compounds, as well as comprehensive evaluation of their clinical efficacy and safety, are essential next steps toward their potential integration into clinical practice.

The inhibition of biofilm activity of bisQACs with varying alkyl chain lengths and linker structures against *Staphylococcus aureus* ATCC 25923 was examined. Compound 2(QC_16_)_3_ exhibited strong inhibition of biofilm activity at higher concentrations, with inhibition exceeding 90% at both 100 and 50 µg/mL. At 25 µg/mL, inhibition slightly decreased to >80%, while a more pronounced decline was observed at 12.5 µg/mL (11%) and 6.25 µg/mL (>2%), indicating reduced efficacy at lower concentrations.

Compound 2(QC_14_)_6_ also showed pronounced activity at 100 µg/mL (>90%) and remained effective at 50 µg/mL. However, a sharp drop to 34% was observed at 25 µg/mL, with further reductions at 12.5 µg/mL (15%) and 6.25 µg/mL (13%). Among the tested derivatives, 2(QC_16_)_6_ demonstrated the most stable inhibition profile, with 95% inhibition at 100 µg/mL and sustained activity >90% at 50 µg/mL. The activity remained >80% at 25 µg/mL but declined to >30% at 12.5 µg/mL and to 6% at 6.25 µg/mL, suggesting superior performance at intermediate concentrations relative to other analogs.

The commercial QAC BAB (benzyldodecyldimethylammonium bromide) exhibited consistently high biofilm inhibition (>74%) across all tested concentrations, suggesting a concentration-independent effect. CPC (cetylpyridinium chloride) showed a concentration-dependent pattern, with maximal inhibition (>70%) at 25 µg/mL and markedly lower activity (>30%) at 12.5 µg/mL. Cefotaxime (CFX) showed moderate biofilm inhibition activity, achieving 62% inhibition at 50 µg/mL but lacking significant efficacy at lower concentrations. These results highlight BAB as a promising biofilm inhibition agent with stable performance, while CPC and CFX display reduced activity at subinhibitory concentrations. These data support further exploration of these compounds in the context of biofilm-associated *S. aureus* infections. All tested bisquaternary derivatives exhibited notable biofilm inhibition activity against *L. monocytogenes*, with 2(QC_16_)_3_ emerging as the most effective across all concentrations. Its sustained high-level inhibition even at low concentrations highlights its potential for further development. In contrast, 2(QC_16_)_4_ and particularly 2(QC_16_)_6_ showed a concentration-dependent decrease in activity, suggesting that structural modifications can significantly influence biofilm inhibition performance. These findings underscore the importance of structural optimization and warrant further investigation into the mechanisms underlying their activity.

Among the tested bisquaternary derivatives, compound 2(QC_16_)_3_ exhibited the highest biofilm inhibition efficacy. Nearly complete inhibition was achieved at 100 µg/mL (97%), and activity remained exceptionally high at lower concentrations: 94% at 50 µg/mL, 94% at 25 µg/mL, and 93% at 12.5 µg/mL. Even at the lowest tested concentration (6.25 µg/mL), inhibition was maintained at 91%, indicating robust and concentration-independent biofilm inhibition activity. Compound 2(QC_16_)_4_ also demonstrated strong biofilm inhibition activity, albeit slightly less effective than 2(QC_16_)_3_. At 100 µg/mL, inhibition reached 92%, and it remained high (>90%) at 50 and 25 µg/mL. However, activity declined at lower concentrations—dropping to >70% at 12.5 µg/mL and >50% at 6.25 µg/mL—highlighting reduced potency at subinhibitory levels. Compound 2(QC_16_)_6_ exhibited excellent activity, with 89% inhibition observed at 100 µg/mL. Although a concentration-dependent decline was noted—88% at 50 µg/mL, 81% at 25 µg/mL, 69% at 12.5 µg/mL, and 40% at 6.25 µg/mL—the compound maintained substantial inhibitory effects even at lower concentrations.

Biofilm inhibition by commercially available antimicrobials—cefotaxime (CFX), cetylpyridinium chloride (CPC), and benzyldimethyldodecylammonium bromide (BAB)—against *L. monocytogenes* was investigated. Cefotaxime demonstrated low to moderate biofilm inhibition activity, with inhibition values >20% at 100 and 50 µg/mL. However, no substantial improvement was observed at lower concentrations: 6% at 25 µg/mL and similarly low values at 12.5 µg/mL, indicating limited efficacy of this *β*-lactam antibiotic against established biofilms. CPC exhibited significantly greater activity compared to CFX, achieving 45% inhibition at 100 µg/mL and 39% at 50 µg/mL. Nevertheless, its efficacy declined at lower concentrations, with 19% inhibition at 25 µg/mL and 15% at 12.5 µg/mL.

BAB showed a comparable profile to CPC but with slightly more stable performance at intermediate concentrations. At 100 µg/mL, inhibition reached 44%, remaining consistent at 50 µg/mL. At 25 and 12.5 µg/mL, BAB maintained 20% and comparable values, respectively, suggesting improved performance over CPC at lower concentrations. Collectively, these results highlight the superior biofilm inhibition activity of 2(QC_16_)_3_ compared to both other bisquaternary analogs and commercial antimicrobials. The data suggest that structural refinement of bisquaternary ammonium compounds could facilitate the development of effective strategies for controlling *L. monocytogenes* biofilm-related infections.

### 2.6. Time-Dependent Activity of bisQACs

Time-kill assays are a standard approach for evaluating the bactericidal dynamics of antimicrobial agents by monitoring microbial viability over defined incubation intervals. Compounds exerting ≥ 99.99% reduction in viable cell count (≥3 log_10_ CFU/mL) within 24 h are classified as bactericidal, whereas bacteriostatic agents suppress growth without inducing cell death [52]. Due to their pronounced membrane-disruptive properties, quaternary ammonium compounds (QACs) are predominantly bactericidal, inducing rapid membrane destabilization and irreversible cell lysis [44]. While some QACs exhibit intracellular activity—potentially targeting DNA or proteins—the molecular basis of these effects remains incompletely understood and warrants further investigation [53,54].

**Figure 6:**
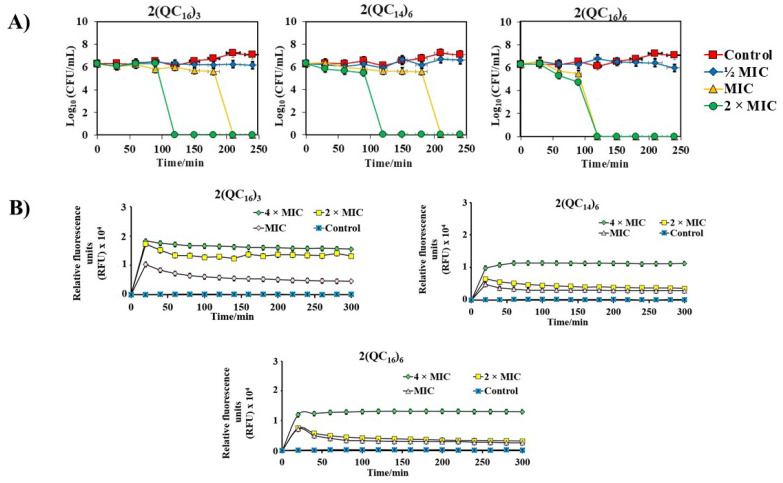
(**A**) Time-dependent reduction of *Staphylococcus aureus* ATCC 25923 viable cells, expressed as log_10_ CFU/mL, monitored via plate counting during a 4 h exposure to ½ MIC, MIC, and 2 × MIC concentrations of bisquaternary ammonium compounds 2(QC_16_)_3_, 2(QC_14_)_6_, and 2(QC_16_)_6_. (**B**) Kinetic analysis of membrane integrity disruption measured by propidium iodide (PI) uptake using spectrofluorimetry in *Staphylococcus aureus* ATCC 25923 treated with 4 × MIC, 2 × MIC, and MIC of the tested compounds compared to untreated controls. Fluorescence intensity was recorded at defined time points over a 6 h incubation period.

The investigated bisquaternary ammonium compounds (bisQACs) based on 3-aminoquinuclidine exert a bactericidal effect through a membrane-disruptive mechanism, achieving complete eradication of *Staphylococcus aureus* ATCC 25923 within 24 h (Figure 6A). However, time-kill kinetics revealed notable differences in the rate of action among tested derivatives, largely influenced by alkyl chain length and linker flexibility. Compounds 2(QC_16_)_3_ and 2(QC_14_)_6_ exhibited rapid bactericidal activity within 3 h, with complete killing observed at 2 × MIC after only 2 h. The enhanced efficacy of longer alkyl chains suggests stronger hydrophobic interactions with the bacterial membrane, leading to faster destabilization.

Among all tested compounds, 2(QC_16_)_6_ showed the most potent and rapid bactericidal activity, eliminating viable cells within 2 h. This highlights the critical role of linker length and flexibility in facilitating optimal orientation of quaternary centers within the lipid bilayer, thereby enhancing membrane disruption. These findings underscore the synergistic impact of alkyl chain length and linker architecture in tuning bactericidal performance, offering valuable insight for the design of next-generation fast-acting antimicrobial agents.

### 2.7. Spectrofluorimetric Analysis of Propidium Iodide (PI) Uptake Assay

The results presented in Figure 6B demonstrate that exposure of bacteria to MIC, 2 × MIC, and 4 × MIC concentrations of bisquaternary ammonium compounds (bisQACs) induces a time-dependent increase in PI uptake, reflecting compromised bacterial membrane integrity. This is evidenced by a significant rise in fluorescence intensity compared to the untreated control, which showed negligible PI uptake and preserved membrane integrity throughout the 300 min observation period. At higher concentrations, particularly at 4 × MIC, a rapid fluorescence increase occurred almost immediately, indicating swift membrane disruption leading to cell death or severe injury.

Among the bisquaternary ammonium compounds tested—2(QC_16_)_3_, 2(QC_14_)_6_, and 2(QC_16_)_6_—the fastest membrane-disruptive effects were observed for those featuring a six-carbon linker. This highlights the critical role of the spatial distance between the two positively charged quaternary ammonium centers in modulating antibacterial efficacy. Shorter linkers may increase molecular rigidity, thereby limiting effective interaction with the bacterial membrane, while longer linkers enhance molecular flexibility, allowing improved conformational adaptation to destabilize the lipid bilayer. Nevertheless, excessively long linkers can diminish activity by disrupting the structural organization necessary for optimal membrane binding. These findings, together with the concentration-dependent membrane disruption observed in *Staphylococcus aureus* ATCC 25923, underscore the importance of linker length and compound concentration in designing potent bisQAC antimicrobials. Further mechanistic studies are warranted to optimize these parameters for enhanced therapeutic application.

**Figure 7:**
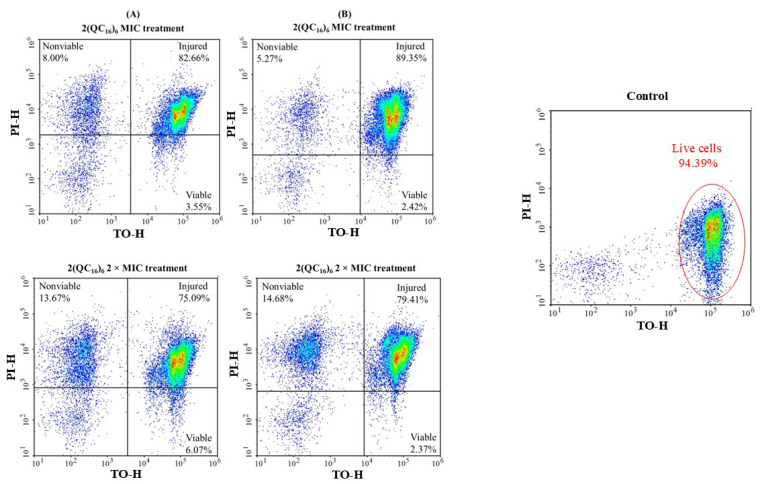
Flow cytometry dot plots of viable, injured, and dead *Staphylococcus aureus* ATCC 25923 cells stained with thiazole orange (TO) and propidium iodide (PI), at the beginning of the experiment (**A**) and after 4 h (**B**) of treatment with 2(QC_16_)_6_ at MIC and 2 × MIC concentrations.

To precisely characterize the antimicrobial activity of the synthesized bisquaternary ammonium compounds (bisQACs) against *Staphylococcus aureus* ATCC 25923, flow cytometry was employed to assess membrane integrity, alongside classical colony-forming unit (CFU) enumeration as a functional viability assay. All compounds were tested at concentrations corresponding to the MIC and 2 × MIC, with compound 2(QC_16_)_6_ selected as the representative bisQAC due to its pronounced and rapid disruption of the bacterial membrane of *S. aureus* ATCC 25923 (Figure 7). At the onset of treatment, over 80% of cells exhibited signs of membrane damage (TO+/PI+), while fewer than 5% remained fully viable (TO+/PI−). After 4 h of incubation, the proportion of viable cells further declined, with injured and dead populations dominating. Quadrant analysis (TO and PI staining) allowed discrimination between live (TO+/PI−), injured (TO+/PI+), and dead (TO−/PI+) cells, and debris (TO−/PI−), revealing a clear shift toward irreversible membrane damage over time.

Although the majority of the population remained in a sub-lethally injured state (TO+/PI+), the classical CFU assay confirmed complete loss of culturability within just 2 h of exposure to 2(QC_16_)_6_, validating its rapid and potent bactericidal effect. These findings highlight the importance of combining physiological (flow cytometry) and functional (CFU-based) assays when evaluating antimicrobial mechanisms of action. While flow cytometry provides a sensitive snapshot of membrane integrity and metabolic activity, it cannot reliably predict long-term survival and replicative capacity. In contrast, the CFU method exclusively quantifies cells capable of proliferation, which is why it remains the gold standard for assessing bactericidal activity.

The results are consistent with the known mechanism of bisQACs, which act through physicochemical interactions with the lipid bilayer, compromising membrane permeability and structural cohesion [44]. This leads to cytoplasmic leakage, osmotic imbalance, and irreversible cellular damage. Overall, the rapid onset of membrane disruption in *S. aureus* ATCC 25923, the dominance of non-viable populations in flow cytometry, and the complete loss of colony-forming ability strongly support the conclusion that bisQACs—particularly 2(QC_16_)_6_—exert a potent bactericidal effect against this pathogenic species.

### 2.8. Cytotoxicity of BisQACs

The cytotoxicity of quaternary ammonium compounds (QACs) remains a critical limitation to their broader biomedical and environmental application [55]. In recent years, research has increasingly focused on the development of structurally refined and environmentally degradable QAC derivatives with reduced toxicity toward non-target organisms [56]. Given their widespread use as disinfectants and antiseptics, ensuring their safety for potential human and veterinary use is essential [57]. Therefore, the cytotoxicity of selected bisQAC candidates was evaluated using healthy human cell lines: HEK293 (human embryonic kidney cells) and RPE1 (retinal pigment epithelial cells). The results were benchmarked against two commercially used QACs—benzyldodecyldimethylammonium bromide (BAB) and cetylpyridinium chloride (CPC).

**Figure 8:**
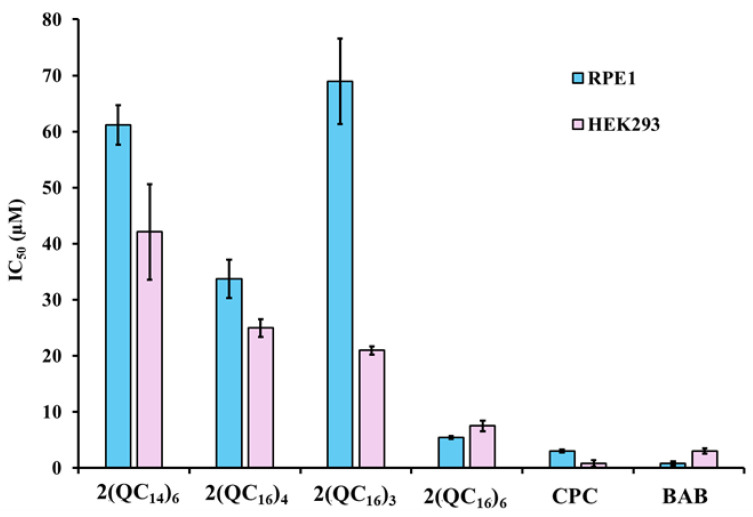
Cytotoxicity of bisQACs toward healthy mammalian cell lines: retinal pigment epithelial cells (RPE1) and human embryonic kidney cells (HEK293). Half-maximal inhibitory concentrations (IC_50_) were determined and compared to those of commercial quaternary ammonium compounds—cetylpyridinium chloride (CPC) and benzyldodecyldimethylammonium bromide (BAB).

As shown in Figure 8, the tested compounds exhibited distinct toxicity profiles, demonstrating that the longer the alkyl linker, the greater the cytotoxicity. In RPE1 cells, compound 2(QC_14_)_6_ displayed moderate cytotoxicity with an IC_50_ value of 61 μM, followed by 34 μM for 2(QC_16_)_4_, 69 μM for 2(QC_16_)_3_, and 5 μM for 2(QC_16_)_6_. A comparable trend was observed in HEK293 cells, where IC_50_ values were 42 μM for 2(QC_14_)_6_, 25 μM for 2(QC_16_)_4_, 21 μM for 2(QC_16_)_3_, and 7 μM for 2(QC_16_)_6_. Interestingly, 2(QC_16_)_3_ exhibited lower toxicity in RPE1 than 2(QC_16_)_4_, which may reflect differences in membrane composition or compound uptake between the two cell types.

Both CPC and BAB exhibited high toxicity across both cell types (Figure 8), serving as benchmarks for commercially used QACs with known membrane-disruptive profiles. These findings underscore a general structure–toxicity relationship, where increased hydrophobicity due to extended alkyl chains enhances membrane-disruptive potential not only against bacterial cells but also in mammalian systems. Among the tested compounds, 2(QC_16_)_6_ showed the highest cytotoxicity in both cell lines, emphasizing the importance of carefully balancing antimicrobial potency with host cell safety in the design of novel bisQAC-based disinfectants or therapeutics. This highlights the need to define a clear therapeutic window, where antibacterial efficacy can be achieved without compromising mammalian cell viability. Future optimization efforts could focus on structural modifications, such as adjusting alkyl chain length or introducing polar substituents, to fine-tune lipophilicity and membrane selectivity. In this way, the balance between potency and safety could be improved, supporting the development of bisQAC derivatives with more favorable pharmacological profiles.

To better evaluate the therapeutic potential of the tested compounds, we calculated the selectivity index (SI), defined as the ratio between the cytotoxic concentration (IC_50_) in mammalian cells (RPE1 and HEK293) and the antibacterial potency (MIC) against *Staphylococcus aureus* ATCC 25923 (Table 2).

Among the tested derivatives, 2(QC_16_)_3_ and 2(QC_14_)_6_ displayed the most favorable profiles, with SI values above unity in RPE1 cells (1.82 and 1.61, respectively), indicating a degree of selectivity towards bacterial cells over mammalian cells. In HEK293, only 2(QC_14_)_6_ maintained an SI slightly greater than 1 (1.11), whereas the remaining compounds exhibited SI values below 1, suggesting limited safety margins.

In contrast, 2(QC_16_)_6_, despite showing potent antibacterial activity (MIC = 9 µM), had relatively low SI values (0.56 in RPE1 and 0.78 in HEK293), reflecting a narrow therapeutic window. The commercial agents CPC (cetylpyridinium chloride) and BAB (benzyldodecyldimethylammonium bromide) also exhibited poor selectivity (SI < 1), consistent with their known cytotoxicity profiles.

Overall, the SI analysis highlights the importance of structural variation in modulating the balance between efficacy and cytotoxicity within this compound series. Notably, 2(QC_16_)_3_ and 2(QC_14_)_6_ emerge as the most promising candidates for further optimization, as they combine antibacterial activity with a more favorable selectivity index compared to both other quaternary derivatives and commercial compounds.

The cytotoxicity of QACs is primarily driven by their ability to interact with and disrupt mammalian cell membranes. Their amphiphilic structure, comprising a positively charged quaternary ammonium headgroup and hydrophobic alkyl chains, enables insertion into the phospholipid bilayer, causing loss of membrane integrity, leakage of cytoplasmic content, and eventual cell death [44]. The observed correlation between longer alkyl chains and increased cytotoxicity in both RPE1 and HEK293 cells supports this mechanism, as higher lipophilicity enhances membrane partitioning and destabilization [58]. In addition to direct membrane disruption, several studies have reported that QACs may trigger mitochondrial dysfunction, generation of reactive oxygen species (ROS), and apoptotic signaling pathways at sublytic concentrations [59,60]. These secondary mechanisms may contribute to the differences observed between the two tested cell types, such as the lower toxicity of 2(QC_16_)_3_ in RPE1 compared to HEK293. Altogether, the cytotoxic profiles of bisQAC derivatives likely reflect a combination of direct membrane-lytic activity and downstream stress responses, consistent with the known mode of action of commercial agents such as CPC and BAB [61].

## 3. Structure-Activity Relationship (SAR)

### In Silico Molecular Docking Study of bisQACs Binding to the QacR Regulatory Protein

The expression of efflux systems targeting quaternary ammonium compounds (QACs) in multidrug-resistant bacteria is regulated by the transcriptional repressor QacR, a key modulator of gene expression for multidrug efflux pumps [62]. Upon binding of small-molecule effectors, such as QACs, QacR undergoes conformational changes that relieve repression and activate the transcription of efflux-related genes, thereby contributing to the development of antimicrobial resistance [63].

Wuest and colleagues previously demonstrated that multiple quaternization of antimicrobial scaffolds can reduce their ability to interact with QacR, ultimately decreasing the induction of efflux mechanisms [64]. In agreement with these findings, our data show that bisquaternary ammonium salts (bisQACs) exhibit significantly weaker binding affinities toward QacR protein compared to commercial bisQAC octenidine dihydrochloride (OCT), as evidenced by their higher calculated binding free energies (ΔG_bind_).

This reduced interaction with QacR implies a diminished potential for triggering efflux pump expression [65]. Thus, bisQACs molecules emerge as promising antimicrobial candidates with minimized risk for resistance development. These findings provide a mechanistic foundation for the rational design of next-generation QAC-based antimicrobials that retain efficacy while limiting resistance induction through transcriptional regulation.

**Figure 9:**
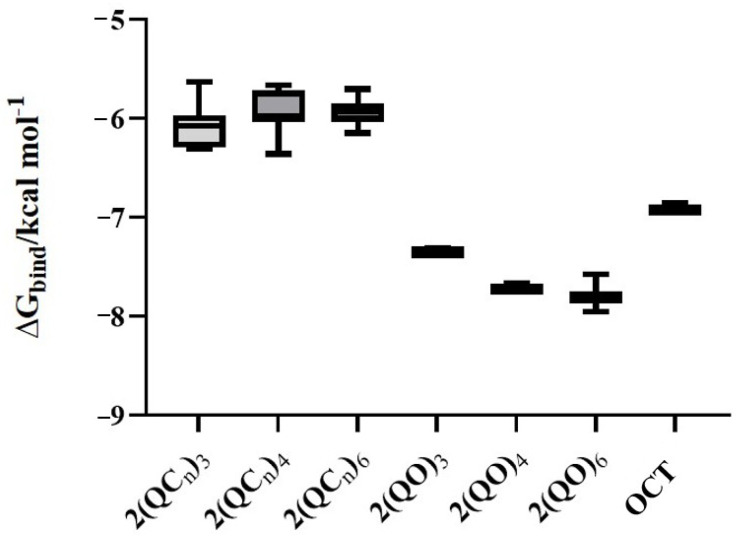
Box-and-whisker plot of calculated ΔG_bind_ values for bisQAC analogs (*n* = 12 C, 14 C, and 16 C) interacting with QacR protein.

As shown in Figure 9, modifications in alkyl chain length and linker length significantly influence the binding affinity of quaternary ammonium compounds toward the transcriptional regulator QacR. In general, compounds bearing longer alkyl chains (e.g., C_16_) exhibit markedly weaker binding affinities compared to their shorter-chain analogs (e.g., C_12_), a trend particularly evident in molecules with the shortest linker (*n* = 3). Given that stronger QacR binding leads to derepression of efflux pump genes, the reduced interaction of these long chain bisQAC derivatives with QacR may be considered a favorable feature for preventing the activation of bacterial resistance mechanisms.

Our findings further suggest that replacing the hydrophobic alkyl chain with a carbonyl-containing fragment substantially increases the affinity of the compounds for QacR, supporting the hypothesis that more polar substituents enhance interactions with regulatory domains of the protein. Experimental validation of these observations is part of our planned future research, which will provide further insight into the relationship between compound structure and efflux regulator binding.

Octenidine dihydrochloride (OCT), a reference antiseptic, exhibits an intermediate binding affinity toward QacR. Its affinity is notably higher than that of the newly synthesized 2(QCn) bisquaternary ammonium salts (bisQACs), yet lower than that of the more strongly interacting bisquaternary salts derived from quinuclidin-3-one.

Considering QacR’s role as a transcriptional repressor of efflux systems, high-affinity ligands may promote gene derepression, facilitating rapid efflux of antimicrobial agents and potentially compromising their intracellular stability and bactericidal efficacy. In contrast, compounds such as 2(QC_n_), which exhibit weaker binding to QacR, may avoid triggering efflux mechanisms and thus maintain prolonged intracellular activity. This feature renders them attractive candidates for further development as antimicrobial agents with a reduced propensity to induce bacterial resistance.

The crystal structure of the multidrug-binding transcriptional repressor QacR (PDB ID: 1JUP) was chosen because it is a well-characterized model protein for studying the binding of structurally diverse cationic and hydrophobic ligands. Given that bisQAC compounds share chemical features with substrates known to interact with QacR, this protein provides a relevant structural framework to rationalize the observed biological activity.

**Figure 10:**
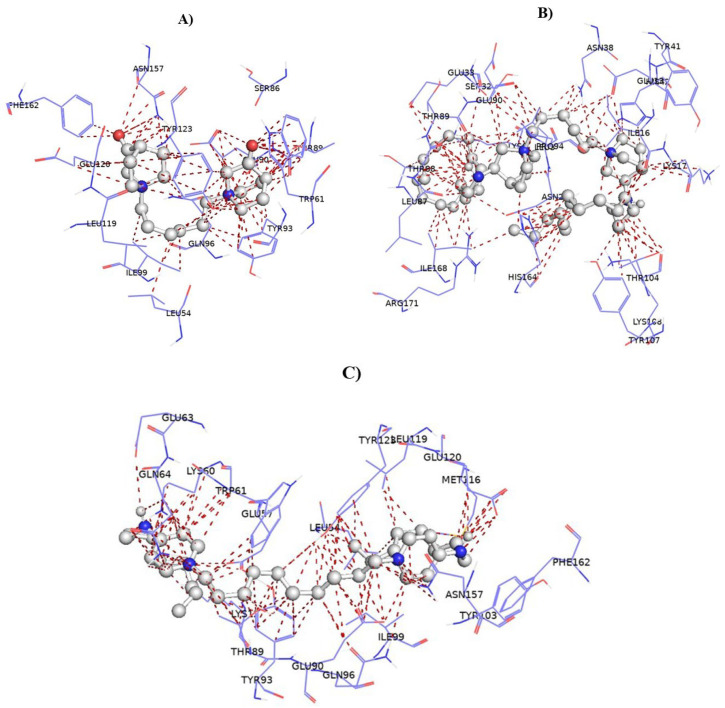
Molecular simulation of the QacR protein complexed with bisQAC compounds within the hydrophobic binding pocket: (**A**) 2(QO)_6_, (**B**) 2(QC_16_)_6_, and (**C**) OCT. Carbon atoms are shown in grey, nitrogen in blue, and oxygen atoms and interactions in red.

The hydrophobic binding pocket of QacR exhibits notable structural plasticity, enabling the recognition and accommodation of structurally diverse quaternary ammonium compounds, including bisQACs (Figure 10). This adaptability arises from conformational flexibility in side chains of key residues and the inducible nature of the multidrug-binding site [66,67].

Binding of bisQACs is primarily driven by a combination of non-covalent interactions, including electrostatic interactions between the quaternary nitrogen atoms and acidic residues (e.g., Asp, Glu), hydrophobic contacts between the long alkyl chains and nonpolar residues (e.g., Leu, Val, Phe), and potential π–cation interactions involving aromatic residues such as Tyr or Phe [68]. Additional van der Waals forces further stabilize the ligand within the cavity [69].

This combination of interactions underlies the broad substrate specificity of QacR and its ability to respond to a range of cationic antimicrobial agents by triggering transcriptional activation of efflux pump genes.

## 4. Materials and Methods

### 4.1. Synthesis

#### 4.1.1. Deprotonation of Quinuclidin-3-One Hydrochloride

Quinuclidin-3-one was efficiently prepared in high yields (>90%) by treating commercially available quinuclidin-3-one hydrochloride (200 mg, 1.24 mmol; Sigma-Aldrich, St. Louis, MO, USA) with a saturated aqueous potassium hydroxide solution (7.13 mmol in 0.4 mL H_2_O, Gram-mol, Zagreb, Croatia). The reaction mixture was then subjected to liquid–liquid extraction using chloroform (10 × 2 mL, GPR RECTAPUR®, VWR Chemicals, Radnor, PA, USA). The combined organic layers were dried over anhydrous potassium carbonate (K_2_CO_3_, Gram-mol, Zagreb, Croatia), filtered, and concentrated under reduced pressure. To completely remove residual moisture, the product was stored under reduced pressure over anhydrous calcium chloride (CaCl_2_). The purified quinuclidin-3-one was then used as a key precursor for synthesizing new quaternary ammonium derivatives.

#### 4.1.2. Synthesis of bisQACs of Quinuclidin-3-One

A stoichiometric amount of quinuclidin-3-one was dissolved in anhydrous acetone, followed by the addition of the chosen linker—1,3-dibromopropane (Aldrich Chemie GmbH, Steinheim, Germany), 1,4-dibromobutane (Aldrich Chemistry, Merck KGaA, Darmstadt, Germany), or 1,6-dibromohexane (Alfa Aesar, Haverhill, MA, USA)—in a 1:2 molar ratio favoring quinuclidin-3-one. To maintain an inert environment, the quaternization reaction was carried out under a nitrogen atmosphere using a nitrogen-filled balloon. The mixture was stirred at room temperature for 24 h, with reaction progress monitored by thin-layer chromatography (TLC) on alumina, using a chloroform:methanol (1:1) solvent system. After completion, the solvent was removed under reduced pressure using a rotary evaporator. The resulting white powder was washed with dry diethyl ether to obtain the purified quaternized product.

For the reductive amination, a measured amount of bisquaternized quinuclidin-3-one was dissolved in methanol along with the chosen linker. An excess of the corresponding amines—dodecylamine (Sigma Aldrich, St. Louis, MO, USA), tetradecylamine (Acros Organics, Geel, Belgium), or hexadecylamine (Merck, Darmstadt, Germany)—was added in a 1:2 molar ratio to favor the amine. Then, a stoichiometric amount of sodium cyanoborohydride (NaBH_3_CN), dissolved in methanol, was introduced to the mixture. The reaction was carried out under reflux with continuous stirring for 4 days. Progress was monitored using thin-layer chromatography (TLC) on alumina plates, employing a solvent system of dichloromethane and methanol (9:1). After completion, the solvent was removed under reduced pressure using a rotary evaporator. The resulting sticky precipitate was washed with dry diethyl ether to obtain the target compound with the desired purity.

The resulting bisQAC variants, along with their corresponding IUPAC names and yields, are summarized below. ^1^H NMR and ^13^C NMR spectra of the newly synthesized compounds were recorded in MeOH-d_4_ or DMSO-d_6_ solution on a Bruker Avance III HD 400 MHz/54 mM Ascend spectrometer (Bruker 550 Optics Inc, Billerica, MA, USA). Chemical shifts are given in ppm downfield from tetramethyl silane (TMS).

1,1′-(Propane-1,3-diyl) bis(3-oxoquinuclidinium) dibromide 2(QO)_3_: (*η* = 96%)

^1^H NMR (400 MHz, MeOD-*d*_4_) *δ*/ppm 1.91–2.07 (m, 4 H) 2.11–2.29 (m, 5 H) 2.30–2.40 (m, 5 H) 3.33–3.62 (m, 14 H); ^13^C NMR (100 MHz, MeOD-*d*_4_) *δ*/ppm 17.5 (C3′) 21.2 (C5) 21.5 (C7) 26.3; 29.5; 31.0 (C4) 55.6; 55.7; 55.8; 56.0; 61.6 (C8) 64.1 (C2) 201.1 (C=O).

1,1′-(Butane-1,4-diyl) bis(3-oxoquinuclidinium) dibromide 2(QO)_4_: (*η* = 93%)

^1^H NMR (400 MHz, MeOD-*d*_4_) *δ*/ppm 1.91–2.07 (m, 4 H) 2.11–2.29 (m, 5 H) 2.30–2.40 (m, 5 H) 3.33–3.62 (m, 14 H); ^13^C NMR (100 MHz, MeOD-*d*_4_) *δ*/ppm 17.5 (C3′) 19.8 (C4′) 21.2 (C5) 21.5 (C7) 26.3; 29.5; 31.0 (C4) 55.6; 55.7; 55.8; 56.0; 61.6 (C8) 64.1 (C2) 201.1 (C=O).

1,1′-(Hexane-1,6-diyl) bis(3-oxoquinuclidinium) dibromide 2(QO)_6_: (*η* = 96%)

^1^H NMR (400 MHz, DMSO-*d*_6_) *δ*/ppm: 1.26–1.38 (m, 4 H, H3′, H4′) 1.67–1.79 (m, 4 H, H2′, H5′) 2.03–2.14 (m, 4 H, H5) 2.21–2.33 (m, 4 H, H7) 2.67–2.75 (m, 2 H, H4) 3.36–3.44 (m, 4 H, H1′, H6′) 3.55–3.65 (m, 4 H, H6) 3.65–3.77 (m, 4 H, H8) 4.25–4.30 (m, 4 H, H2); ^13^C NMR (100 MHz, DMSO-*d*_6_) *δ*/ppm: 20.7 (C3′, C4′) 21.1 (C5) 21.2 (C7) 24.9; 25.2; 27.0; 31.8; 35.0; 36.8 (C4) 53.9 (C1′, C6′) 62.9 (C6) 63.1 (C8) 64.8 (C2) 203.2 (C=O).

(±)-1,1′-(Propane-1,3-diyl) bis(3-(dodecylamino)quinuclidinium) dibromide 2(QC_12_)_3_: (*η* = 92%)

^1^H NMR (400 MHz, DMSO-*d*_6_) *δ*/ppm: 0.85 (t, *J* = 6.7 Hz, 6 H, H12″) 1.24 (s, 36 H) 1.33–1.44 (m, 4 H, H2″) 1.69–1.85 (m, 4 H, H1″) 1.86–1.99 (m, 2 H) 2.02–2.09 (m, 2 H) 2.13 (br. s., 2 H) 2.32–2.48 (m, 2 H) 2.54–2.70 (m, 2 H) 2.85–3.13 (m, 4 H) 3.13–3.26 (m, 4 H) 3.27–3.48 (m, 10 H) 3.58–3.75 (m, 2 H) 3.83–3.88 (m, 1 H) 4.03–4.13 (m, 2 H); ^13^C NMR (100 MHz, DMSO-*d*_6_) *δ*/ppm: 14.4 (C12″); 15.5; 15.5; 17.3; 18.9; 19.6; 20.8; 20.9; 22.1; 25.7; 26.3; 26.8; 26.9; 28.66; 28.69; 28.96; 28.99; 29.0; 29.8; 31.3; 37.0; 46.1; 46.5; 53.2; 54.2; 54.7; 62.6 (C2); 63.4 (C3).

(±)-1,1′-(Propane-1,3-diyl) bis(3-(tetradecylamino)quinuclidinium) dibromide 2(QC_14_)_3_: (*η* = 86%)

^1^H NMR (400 MHz, DMSO-*d*_6_) *δ*/ppm: 0.85 (t, *J* = 6.7 Hz, 6 H, H14″) 1.19–1.30 (m, 44 H) 1.35–1.41 (m, 4 H) 1.70–1.85 (m, 6 H) 1.89–1.97 (m, 4 H) 2.03–2.08 (m, 2 H) 2.10–2.21 (m, 4 H) 2.84–2.97 (m, 2 H) 2.99–3.11 (m, 4 H) 3.13–3.26 (m, 8 H) 3.37–3.47 (m, 2 H) 3.59–3.75 (m, 3 H) 3.84–3.88 (m, 1 H) 4.05–4.12 (m, 3 H); ^13^C NMR (100 MHz, DMSO-*d*_6_) *δ*/ppm: 14.4 (C14″); 16.0; 17.8; 21.3; 21.4; 22.5; 22.6; 27.3; 29.2; 29.47; 29.51; 30.04; 30.14; 30.20; 31.76; 37.50; 39.38; 39.58; 39.8; 40.0; 40.2; 40.4; 40.6; 40.9; 53.1; 53.7; 54.7 (C4); 60.1; 62.1; 63.1 (C2); 63.8 (C3).

(±)-1,1′-(Propane-1,3-diyl) bis(3-(hexadecylamino)quinuclidinium) dibromide 2(QC_16_)_3_: (*η* = 35%)

^1^H NMR (400 MHz, MeOD-*d*_4_) *δ*/ppm: 0.76–0.84 (m, 6 H, H16″) 1.15–1.29 (m, 54 H) 1.35–1.46 (m, 2 H) 1.74–1.92 (m, 4 H) 1.93–2.06 (m, 2 H) 2.07–2.12 (m, 1 H) 2.13–2.29 (m, 2 H) 2.37–2.51 (m, 1 H) 3.00–3.17 (m, 4 H) 3.23 (s, 1 H) 3.27 (br. s., 1 H) 3.33–3.52 (m, 3 H) 3.52–3.63 (m, 1 H) 3.65–3.78 (m, 1 H) 4.07–4.19 (m, 1 H); ^13^C NMR (100 MHz, MeOD-*d*_4_) *δ*/ppm: 14.4 (C16″); 18.8; 21.6; 22.4; 22.4; 23.7; 25.2; 25.6; 28.5; 30.4; 30.5; 30.67; 30.71; 30.74; 30.77; 30.83; 33.1; 39.0; 40.5; 55.2; 56.4 (C4); 56.6; 61.6; 62.9; 63.5; 65.4 (C3); 70.9 (C2).

(±)-1,1′-(Butane-1,4-diyl) bis(3-(dodecylamino)quinuclidinium) dibromide 2(QC_12_)_4_: (*η* = 98%)

^1^H NMR (400 MHz, MeOD-*d*_4_) *δ*/ppm: 0.82–0.96 (m, 6 H, H12″) 1.11 (m, 4 H) 1.25–1.40 (m, 38 H) 1.42–1.63 (m, 4 H) 1.76–1.84 (m, 4 H) 1.86–1.98 (m, 4 H) 2.02–2.13 (m, 2 H) 2.16–2.40 (m, 4 H) 2.97–3.20 (m, 6 H) 3.37–3.51 (m, 8 H) 3.63–3.77 (m, 2 H) 4.18–4.25 (m, 1 H); ^13^C NMR (100 MHz, MeOD-*d*_4_) *δ*/ppm: 14.4 (C12″) 18.8; 20.4; 22.4; 23.7; 28.0; 28.5; 30.4; 30.5; 30.7; 30.8; 31.0; 33.1; 54.2 (C4) 55.0; 56.2; 64.3; 64.7 (C2) 65.4 (C3).

(±)-1,1′-(Butane-1,4-diyl) bis(3-(tetradecylamino)quinuclidinium) dibromide 2(QC_14_)_4_: (*η* = 70%)

^1^H NMR (400 MHz, MeOD-*d*_4_) *δ*/ppm: 0.75–0.84 (m, 6 H, H14″) 1.13–1.30 (m, 44 H) 1.37–1.54 (m, 4 H) 1.66–1.89 (m, 6 H) 1.92–2.04 (m, 2 H) 2.05–2.16 (m, 2 H) 2.19–2.28 (m, 1 H) 2.52–2.63 (m, 2 H) 3.01–3.13 (m, 4 H) 3.25–3.40 (m, 8 H) 3.57–3.62 (m, 1 H) 4.04–4.20 (m, 1 H); ^13^C NMR (100 MHz, MeOD-*d*_4_) *δ*/ppm: 14.4 (C14″) 18.8; 20.4; 22.4; 23.7; 27.9; 30.3; 30.5; 30.52; 30.66; 30.7; 30.71; 30.75; 30.77; 30.8; 32.3; 33.1; 39.0; 42.0; 54.2 (C4) 55.0; 56.2; 64.3; 64.7 (C2) 65.4 (C3).

(±)-1,1′-(Butane-1,4-diyl) bis(3-(hexadecylamino)quinuclidinium) dibromide 2(QC_16_)_4_: (*η* = 77%)

^1^H NMR (400 MHz, MeOD-*d*_4_) *δ*/ppm: 0.85–0.94 (m, 6 H, H16″) 1.24–1.39 (m, 54 H, H2″-H15″) 1.41–1.47 (m, 4 H) 1.48–1.58 (m, 3 H) 1.71–1.83 (m, 4 H) 1.83–1.98 (m, 4 H) 2.02–2.13 (m, 2 H) 2.16–2.20 (m, 1 H) 2.21–2.39 (m, 4 H) 3.09–3.24 (m, 6 H) 3.33–3.49 (m, 7 H) 3.63–3.75 (m, 2 H) 4.18–4.25 (m, 2 H); ^13^C NMR (100 MHz, MeOD-*d*_4_) *δ*/ppm: 14.4 (C16″) 18.8; 20.4; 22.4; 23.7; 27.9; 30.3; 30.4; 30.5; 30.54; 30.6; 30.68; 30.70; 30.72; 30.76; 30.77; 30.79; 32.5; 33.1; 39.0; 42.1; 54.2 (C4) 55.0; 56.2; 64.3; 64.7 (C2) 65.4 (C3).

(±)-1,1′-(Hexane-1,6-diyl) bis(3-(dodecylamino)quinuclidinium) dibromide 2(QC_12_)_6_: (*η* = 96%)

^1^H NMR (400 MHz, MeOD-*d*_4_) *δ*/ppm: 0.80 (t, *J* = 6.8 Hz, 6 H, H12″) 1.13–1.26 (m, 36 H) 1.27–1.48 (m, 12 H) 1.51–1.61 (m, 2 H) 1.63–1.74 (m, 4 H) 1.75–1.91 (m, 4 H) 1.92–2.05 (m, 2 H) 2.06–2.15 (m, 2 H) 2.18–2.28 (m, 2 H) 3.00–3.07 (m, 2 H) 3.07–3.17 (m, 5 H) 3.23–3.44 (m, 8 H) 3.55–3.68 (m, 2 H) 4.08–4.16 (m, 1 H); ^13^C NMR (100 MHz, MeOD-*d*4) *δ*/ppm: 14.4 (C12″) 18.8; 22.4; 22.7; 23.7; 26.8; 27.9; 28.1; 28.5; 30.27; 30.29; 30.35; 30.46; 30.6; 30.66; 30.70; 30.74; 30.76; 30.78; 33.1; 39.0; 54.2 (C4); 54.8; 56.0; 64.6; 65.1 (C2); 65.5 (C3).

(±)-1,1′-(Hexane-1,6-diyl) bis(3-(tetradecylamino)quinuclidinium) dibromide 2(QC_14_)_6_: (*η* = 99%)

^1^H NMR (400 MHz, MeOD-*d*_4_) *δ*/ppm: 0.90 (t, *J* = 6.8 Hz, 6 H, H14″) 1.26–1.36 (m, 48 H) 1.42–1.47 (m, 4 H) 1.51–1.56 (m, 4 H) 1.71–1.82 (m, 4 H) 1.84–1.97 (m, 4 H) 2.15–2.20 (m, 2 H) 3.09–3.24 (m, 8 H) 3.34–3.48 (m, 10 H) 3.64–3.75 (m, 3H) 4.18–4.24 (m, 2 H); ^13^C NMR (100 MHz, MeOD-*d*_4_) *δ*/ppm: 14.4 (C14″) 18.8; 22.4; 22.7; 23.7; 26.8; 27.9; 30.47; 30.51; 30.6; 30.71; 30.76; 30.77; 30.79; 32.1; 33.1; 42.00; 54.3 (C4); 54.8; 56.0; 64.6; 65.2 (C2) 65.5 (C3).

(±)-1,1′-(Hexane-1,6-diyl) bis(3-(hexadecylamino)quinuclidinium) dibromide 2(QC_16_)_6_: (*η* = 58%)

^1^H NMR (400 MHz, MeOD-*d*_4_) *δ*/ppm: 0.86–0.93 (m, 6 H, H16″) 1.23–1.39 (m, 54 H) 1.43–1.46 (m, 4 H) 1.48–1.58 (m, 4 H) 1.71–1.83 (m, 4 H) 1.83–1.98 (m, 4 H) 2.02–2.13 (m, 2 H) 2.16–2.20 (m, 2 H) 2.21–2.39 (m, 4 H) 3.09–3.24 (m, 6 H) 3.33–3.49 (m, 8 H) 3.64–3.75 (m, 2 H) 4.18–4.25 (m, 2 H); ^13^C NMR (100 MHz, MeOD-*d*_4_) *δ*/ppm: 14.4 (C16″); 18.8; 22.4; 22.7; 23.7; 26.8; 27.9; 30.3; 30.3; 30.47; 30.52; 30.66; 30.70; 30.75; 30.77; 30.78; 30.9; 32.2; 33.1; 39.0; 42.0; 54.2 (C4); 54.8; 56.0; 64.6; 65.1 (C2); 65.4 (C3).

### 4.2. Broth Microdilution Assays

#### 4.2.1. Minimum Inhibitory Concentration (MIC)

The antimicrobial efficacy of the synthesized bisquaternary ammonium compounds (bisQACs), specifically quinuclidin-3-one and 3-aminoquinuclidine derivatives, was assessed against a representative panel of Gram-positive and Gram-negative bacterial strains. The Gram-positive panel included *Staphylococcus aureus* ATCC 25923, methicillin-resistant *Staphylococcus aureus* (MRSA, clinical isolate), *Staphylococcus aureus* ATCC 33591, *Bacillus cereus* ATCC 14579, *Listeria monocytogenes* ATCC 7644, and *Enterococcus faecalis* ATCC 29212. The Gram-negative panel comprised *Escherichia coli* ATCC 25922, *Salmonella enterica* (food isolate), and *Pseudomonas aeruginosa* ATCC 27853. All strains were obtained from BioGnost. Minimum inhibitory concentrations (MICs) were determined using the standard broth microdilution method, following the Clinical and Laboratory Standards Institute (CLSI) guidelines (M07-A10: “Methods for Dilution Antimicrobial Susceptibility Testing for Bacteria That Grow Aerobically”) [70]. Fresh Mueller–Hinton broth (Biolife, Graz, Austria) was inoculated with bacterial cultures and incubated at 35 °C or 37 °C with shaking at 220 rpm. Mid-exponential growth phase cultures (OD_600_ = 0.3–0.5) were standardized to 5 × 10^5^ CFU/mL using OD-to-CFU correlations. The inoculated suspensions were aliquoted into 96-well microtiter plates containing a twofold serial dilution series of the test compounds. After overnight incubation at the appropriate temperature, bacterial viability was assessed via a colorimetric assay employing 2-(4-iodophenyl)-3-(4-nitrophenyl)-5-phenyltetrazolium chloride (INT) at a final concentration of 6 mg/mL. The metabolic activity of viable cells reduced INT to a purple formazan product, indicative of microbial growth. A lack of color development was interpreted as inhibition of bacterial proliferation, thereby confirming the bacteriostatic or bactericidal effect of the tested compounds.

#### 4.2.2. Time-Resolved Growth Analysis

The antimicrobial activity of the candidate compounds against *Staphylococcus aureus* ATCC 25923 and *Listeria monocytogenes* ATCC 7644 was further evaluated using a time-resolved growth kinetics assay in 96-well microtiter plates. Overnight bacterial cultures were diluted in fresh, pre-warmed Mueller–Hinton broth (MHB) and incubated at 37 °C with orbital shaking at 220 rpm. Upon reaching the exponential growth phase (OD_600_ = 0.3–0.5), cultures were standardized to a final concentration of 5 × 10^4^ CFU/mL. Aliquots (50 µL) of the standardized inoculum were dispensed into wells containing either no antimicrobial agent (negative control) or the test compounds at concentrations corresponding to ½ MIC and MIC. Bacterial growth was continuously monitored over a 24 h period by measuring absorbance at 595 nm every 10 min using a Bio-Tek EL808 microplate reader (Winooski, VT, USA). Growth kinetics were assessed by comparing the optical density profiles of treated samples against the untreated control. Each experiment was performed independently twice, with all conditions tested in triplicate. Data are presented as the mean values of these replicates.

#### 4.2.3. Potential of Bacterial Resistance Development

To evaluate the potential for bacterial resistance modulation, the minimum inhibitory concentration (MIC) of the candidate compounds was determined in the presence of carbonyl cyanide 3-chlorophenylhydrazone (CCCP), a protonophore known to inhibit ATP synthesis. CCCP disrupts the proton gradient across the bacterial membrane, thereby reducing ATP availability. This loss of membrane potential impairs energy-dependent processes such as efflux pump activity and overall cellular metabolism, potentially sensitizing bacteria to antimicrobial agents. The methicillin-resistant *Staphylococcus aureus* (MRSA) strain ATCC 33591, known to harbor efflux-pump-associated resistance genes, was selected for this study. An overnight culture of MRSA was diluted in fresh Mueller–Hinton broth (MHB) and incubated at 37 °C with shaking at 220 rpm. Once the culture reached the exponential growth phase (OD_600_ = 0.3–0.5), it was further diluted to a final concentration of 5 × 10^5^ CFU/mL using MHB supplemented with 10 µM CCCP. Serial dilutions of the test compounds were prepared in a 96-well microtiter plate containing CCCP-supplemented MHB. A 50 µL aliquot of the standardized bacterial suspension was added to each well. Plates were incubated at 37 °C for 24 h, after which MIC values were determined by visual inspection. The use of CCCP allowed assessment of compound efficacy under conditions of impaired efflux activity, providing insights into possible resistance mechanisms.

#### 4.2.4. Biofilm Inhibition Assay

The ability of the candidate compounds to inhibit biofilm formation was evaluated by determining the minimum biofilm inhibitory concentration (MBIC) against the biofilm-forming strains *Staphylococcus aureus* ATCC 25923 and *Listeria monocytogenes* ATCC 7644. Overnight bacterial cultures were diluted in fresh, pre-warmed Mueller–Hinton broth (MHB) and incubated at 37 °C with constant shaking at 220 rpm. Once the cultures reached the exponential growth phase (OD_600_ = 0.3–0.5), they were standardized to a final concentration of 4 × 10^6^ CFU/mL.

Aliquots (50 µL) of the standardized bacterial suspension were inoculated into 96-well microtiter plates containing the test compounds at final concentrations of 100, 50, 25, and 12.5 µg/mL. Plates were incubated at 37 °C for 24 to 48 h, depending on the bacterial strain. After incubation, the planktonic cells were carefully aspirated to preserve the integrity of the biofilm layer. Wells were gently rinsed with sterile ultrapure Milli-Q water and air-dried at 60 °C for 1 h to fix the biofilms. Biofilm formation was quantified using a crystal violet (CV) staining method. Wells were stained with 100 µL of 0.1% CV solution and incubated at room temperature for 1 h. Excess dye was removed by rinsing with sterile ultrapure Milli-Q water, followed by air-drying. Bound CV was solubilized with 100 µL of 70% ethanol and incubated for 30 min. The solubilized dye was transferred to a fresh plate, and absorbance was measured at 595 nm using a microplate reader (Bio-Tek EL808, Winooski, VT, USA). Absorbance values were directly proportional to biofilm biomass. Untreated bacterial cultures served as positive controls for biofilm formation, while sterile MHB served as the negative control.

#### 4.2.5. Time-Dependent Bactericidal Activity Assessment

To assess the temporal dynamics of bacterial viability upon exposure to the test compounds, a time-kill kinetics assay was performed using the Gram-positive reference strain *Staphylococcus aureus* ATCC 25923. An overnight culture of the strain was subcultured into fresh, pre-warmed Mueller–Hinton broth (MHB) and incubated under aerobic conditions at 37 °C with agitation (220 rpm) until reaching the early exponential growth phase. The bacterial suspension was then standardized to an initial concentration of approximately 5 × 10^5^ CFU/mL. Equal volumes (50 µL) of the standardized inoculum and compound solutions—prepared at final concentrations equivalent to the MIC and 2 × MIC—were dispensed into 96-well microtiter plates. Wells containing untreated bacterial cultures served as growth controls. Immediately after mixing, a 10 µL sample from each well was withdrawn, diluted in 990 µL of MHB, and serially diluted tenfold up to 10^−6^. From each dilution, 20 µL was plated on MHB agar using a sterile loop to determine the initial bacterial count (time zero). The treated plates were incubated at 37 °C, and additional samples were collected at predetermined time points (2, 4, 6, 8, 12, and 24 h) following the same dilution and plating protocol. After 24 h of incubation at 37 °C, colonies were counted, and viable bacterial counts were expressed as log_10_ CFU/mL for each time point. Bactericidal activity was evaluated by plotting the log_10_ CFU/mL over time, providing a dynamic profile of the compound’s killing kinetics.

#### 4.2.6. Assessment of Cell Viability via Flow Cytometry

To gain further insights into the effects of the test compounds on bacterial viability, *Staphylococcus aureus* ATCC 25923 was analyzed using flow cytometry (NovoCyte Advanteon, Agilent Technologies, Santa Clara, CA, USA). Two independent overnight cultures were diluted 1:10 in fresh Mueller–Hinton broth (MHB) and incubated at 37 °C with shaking at 220 rpm until reaching the early exponential growth phase. Cells were then harvested by centrifugation (4500× *g*, 10 min, room temperature). One cell pellet was resuspended in a staining buffer composed of phosphate buffer (pH 7.4) supplemented with 1 mM EDTA and 0.1% Tween 20. The second pellet was treated with absolute ethanol (1:1 *v*/*v*) for 30 min at room temperature to induce membrane permeabilization, followed by centrifugation and washing under identical conditions. This ethanol-treated sample was also resuspended in the same staining buffer. Both preparations were adjusted to a final density of 1 × 10^6^ CFU/mL and served as controls for fluorescence compensation alongside an unstained bacterial population. Bacterial viability was assessed using the BD^TM^ Cell Viability Kit (BD Biosciences, San Jose, CA, USA), which employs thiazole orange (TO) and propidium iodide (PI) for dual fluorescence labeling. Aliquots (50 µL) of the bacterial suspensions in staining buffer were added to 96-well plates containing test compounds at final concentrations equivalent to the MIC and 2 × MIC. Plates were incubated at 37 °C, and at selected points, cell samples were stained with the TO/PI mixture according to the manufacturer’s instructions. Flow cytometric analysis enabled the discrimination of live (TO+/PI−), damaged (TO+/PI+), and dead (TO−/PI+) bacterial populations based on their fluorescence profiles, allowing for a time-resolved evaluation of compound-induced cytotoxicity.

#### 4.2.7. Propidium Iodide (PI) Uptake Assay

To evaluate the membrane-disruptive effects of the test compounds, a propidium iodide (PI) uptake assay was performed using *Staphylococcus aureus* ATCC 25923. An overnight culture was diluted 1:20 in fresh, pre-warmed Mueller–Hinton broth (MHB) and incubated for 1 h at 37 °C with shaking (220 rpm) to reach the early exponential growth phase. Test compounds were freshly prepared in sterile, filtered phosphate-buffered saline (PBS, pH 7.4) at 4 × MIC and serially diluted to final concentrations corresponding to 2 × MIC and MIC. Bacterial cells were harvested by centrifugation (4500× *g*, 10 min, room temperature), washed twice with sterile PBS, and resuspended in PBS to achieve a standardized inoculum of 1 × 10^6^ CFU/mL, confirmed by OD_600_ measurement. Aliquots of 500 µL bacterial suspension were mixed with an equal volume of each compound solution in sterile microcentrifuge tubes. Propidium iodide (PI) stock solution (3 mg/mL) was added to each sample to a final concentration of 5 µM. The mixtures were incubated at 37 °C, and fluorescence was recorded kinetically over a 6 h period using a Tecan Infinite 200 Pro microplate reader (excitation at 536 nm, emission at 617 nm). An increase in fluorescence intensity, indicative of PI uptake due to compromised membrane integrity, was monitored over time to assess the extent of membrane disruption induced by the compounds [71].

#### 4.2.8. Cytotoxicity

The cytotoxic potential of the selected drug candidates was evaluated using the CellTiter 96^®^ AQueous One Solution Cell Proliferation Assay (Promega, Madison, WI, USA), following the manufacturer’s protocol. Human cell lines RPE1 and HEK293 were cultured at 37 °C in a humidified incubator with 5% CO_2_. Test compounds were dissolved in the appropriate growth medium and serially diluted across a range of concentrations in 96-well microtiter plates. Each well was seeded with 5000 cells and incubated for 48 h under standard culture conditions. Following this incubation period, 20 µL of MTS reagent was added to each well, and the plates were further incubated for 3 h to allow for color development. Absorbance was measured at 490 nm using a microplate reader to determine cell viability. Cytotoxicity was quantified as the half-maximal inhibitory concentration (IC_50_), defined as the concentration of compound that reduced cell viability by 50%. Commercial antiseptics cetylpyridinium chloride (CPC) and benzidodecyldimethylammonium bromide (BAB) were included as reference controls. IC_50_ values were calculated using GraFit 6.0 software by plotting compound concentration against absorbance and fitting the data to a sigmoidal dose–response curve.

### 4.3. ADME Drug Properties

#### Quantitative Assessment of Molecular Descriptors

The physicochemical characteristics of the selected compounds were evaluated using the SwissADME web-based platform (http://www.swissadme.ch, accessed on 5 May 2025), a widely utilized computational tool for predicting drug-likeness and key molecular descriptors relevant to pharmacokinetics. Specifically, the calculated octanol–water partition coefficient (cLog*P*) and topological polar surface area (TPSA) were assessed to estimate compound lipophilicity and membrane permeability, respectively. The cLog*P* value reflects a compound’s lipophilicity and plays a crucial role in determining its capacity to cross biological membranes. Higher cLog*P* values indicate increased hydrophobicity, which can enhance membrane permeability and influence pharmacokinetic properties such as absorption, distribution, and oral bioavailability.

The TPSA, also computed using SwissADME, quantifies the total surface area occupied by polar atoms (typically oxygen and nitrogen) and their attached hydrogens. This descriptor is particularly important for evaluating the potential of a compound to permeate cellular membranes, including passage across the blood–brain barrier. Generally, lower TPSA values correlate with improved membrane permeability and, by extension, better bioavailability.

The chemical structures of the tested compounds were constructed using ChemDraw 16.0 (PerkinElmer Informatics, Waltham, MA, USA), a standard tool for molecular design and visualization. The resulting SMILES (Simplified Molecular Input Line Entry System) representations—listed in Table 3—served as input for the SwissADME analyses. SMILES notations offer a concise, machine-readable format that enables efficient computation of molecular descriptors such as cLog*P* and TPSA.

To complement the numerical analysis, TPSA values were visualized using Jmol (http://jmol.sourceforge.net, accessed on 5 May 2025), an open-source molecular visualization program. Jmol provides interactive 3D renderings of molecular structures and their associated polar surface areas, allowing a clearer interpretation of solubility and permeability trends. These visual representations support a deeper understanding of each compound’s pharmacokinetic potential and bioavailability profile.

### 4.4. Molecular Docking of bisQAC Compounds with QacR Protein

The crystal structure of the multidrug-binding transcriptional repressor QacR bound to malachite green (PDB ID: 1JUP) was retrieved from the Protein Data Bank. Prior to docking, all water molecules and heteroatoms were removed to prevent interference with ligand binding. The protein was prepared by adding hydrogen atoms and assigning Kollman charges using AutoDock Tools, ensuring appropriate protonation states for docking. Ligand structures of bisQAC compounds were generated using Open Babel, where 3D conformations were constructed and hydrogens added. Each ligand underwent energy minimization using the MMFF94 force field with 5000 iterations to optimize geometry. The optimized ligands were converted into the PDBQT format required for AutoDock Vina. The docking grid box was centered on the QacR binding site coordinates (X = 121.552, Y = 49.539, Z = 79.909) with dimensions selected to encompass the entire active site, allowing for ligand flexibility during docking. Docking simulations were conducted using AutoDock Vina version 1.2.7, with the exhaustiveness parameter set to 32 to ensure comprehensive sampling of ligand poses. Each bisQAC compound was docked in triplicate to guarantee reproducibility and statistical reliability of predicted binding modes and affinities. The best-scoring docking poses were visualized and analyzed in PyMOL to investigate key interactions between bisQAC ligands and the QacR active site, providing insights into potential molecular determinants of binding affinity.

#### Statistical Analysis

Quantitative binding affinity data for the bisQAC compounds were analyzed using one-way parametric analysis of variance (ANOVA) to evaluate statistically significant differences among groups. Upon obtaining a significant ANOVA result, Tukey’s post-hoc multiple comparisons test was applied to determine specific intergroup differences while controlling the family-wise error rate.

Data distribution and variability were visually represented using box-and-whiskers plots, illustrating the median, interquartile range, and data range. All statistical analyses and graphical representations were performed using GraphPad Prism 10 (GraphPad Software, San Diego, CA, USA). Statistical significance was set at *p* < 0.05. Data are presented as mean ± standard deviation unless otherwise specified.

## 5. Conclusions

This study presents a rationally designed series of quinuclidine-based bisquaternary ammonium compounds (bisQACs) that demonstrate strong potential as next-generation membrane-targeting antimicrobial agents. Through systematic variation of alkyl chain length and linker flexibility, we were able to identify key structure–activity relationships (SARs) governing antimicrobial potency, membrane disruption efficiency, and resistance evasion. Among the synthesized derivatives, compound 2(QC_16_)_6_ exhibited exceptional antimicrobial activity against a broad spectrum of clinically relevant Gram-positive and Gram-negative pathogens, including methicillin-resistant *Staphylococcus aureus* (MRSA), *Listeria monocytogenes*, and *Escherichia coli*, with MIC values in the low micromolar range.

Time-kill kinetics and membrane permeability assays confirmed the rapid, concentration-dependent bactericidal activity of selected bisQACs, primarily mediated through physical disruption of bacterial membranes. Flow cytometry analyses revealed a marked shift from viable to injured and nonviable bacterial populations within hours of treatment, underscoring the compounds’ ability to compromise membrane integrity and induce irreversible damage. Notably, molecular docking studies demonstrated low affinity of these compounds toward the QacR transcriptional regulator, suggesting a diminished capacity to induce efflux-mediated resistance, a common limitation of conventional QACs.

In addition to their antimicrobial potency, the tested bisQACs displayed potent antibiofilm activity, with several derivatives achieving over 90% inhibition of biofilm formation by *S. aureus* and *L. monocytogenes*, even at sub-MIC concentrations. This highlights their capacity to target both planktonic and sessile bacterial populations—a crucial advantage in the context of chronic and device-associated infections. While cytotoxicity testing on HEK293 and RPE1 human cell lines revealed a correlation between increased alkyl chain length and host cell toxicity, select derivatives maintained acceptable IC_50_ values relative to commercial QACs, suggesting a therapeutic window that can be further optimized.

Taking together, these results underscore the promise of quinuclidine-based bisQACs as potent, broad-spectrum antimicrobials with a dual mechanism of action: direct membrane disruption and low potential for resistance induction via efflux modulation. Their strong activity against multidrug-resistant strains and biofilms, combined with favorable physicochemical and ADME properties, supports their candidacy for further preclinical development. Future work will focus on in vivo efficacy evaluation, pharmacokinetic profiling, and formulation strategies to optimize safety and therapeutic indexes for clinical application.

## Figures and Tables

**Table 1:** Minimum inhibitory concentrations (MICs, µM) of newly synthesized bisQACs derived from quinuclidine-3-one.

Compounds	*M*_r_	Gram-Positive Bacteria					Gram-Negative Bacteria			
		*Staphylococcus* *aureus* ATCC 25923	*Staphylococcus* *aureus* ATCC 33591	*Staphylococcus* *aureus* Clinical/MRSA	*Bacillus* *cereus* ATCC 14579	*Listeria* *monocytogenes* ATCC 7644	*Enterococcus* *faecalis* ATCC 29212	*Escherichia* *coli* ATTC 25922	*Salmonella* *enterica* Food Isolate	*Pseudomonas* *aeruginosa* ATTC 27853
		Minimum Inhibitory Concentrations (MIC)/μM								
**2(QO)_3_**	452.23	>75	>75	>75	>75	>75	>75	>75	>75	>75
**2(QO)_4_**	466.26	>75	>75	>75	>75	>75	>75	>75	>75	>75
**2(QO)_6_**	494.31	>75	>75	>75	>75	>75	>75	>75	>75	>75
**2(QC_12_)_3_**	790.94	75	75	>75	>75	38	>75	>75	75	>75
**2(QC_12_)_4_**	804.97	>75	75	>75	>75	75	>75	>75	>75	>75
**2(QC_12_)_6_**	833.02	75	19	>75	>75	38	75	38	>75	>75
**2(QC_14_)_3_**	847.05	75	75	>75	>75	38	>75	>75	>75	>75
**2(QC_14_)_4_**	861.08	>75	75	>75	>75	>75	>75	>75	>75	>75
**2(QC_14_)_6_**	889.32	38	19	75	75	19	38	19	75	>75
**2(QC_16_)_3_**	903.16	38	19	>75	>75	9	>75	>75	>75	>75
**2(QC_16_)_4_**	917.19	75	9	75	>75	9	38	19	75	>75
**2(QC_16_)_6_**	945.24	9	5	38	75	19	38	19	75	>75
**BAB**	384.40	10	25	25	25	10	15	63	50	>125

**Table 2:** Cytotoxicity (IC_50_, µM), antibacterial activity against *Staphylococcus aureus* ATCC 25923 (MIC, µM), and selectivity indices (SI = IC_50_/MIC).

Compounds	IC_50_ (μM)		MIC (μM)	SI (IC_50_/MIC)	
	RPE1	HEK293	*S. aureus*	RPE1	HEK293
**2(QC_14_)_6_**	61	42	38	1.61	1.11
**2(QC_16_)_3_**	69	21	38	1.82	0.55
**2(QC_16_)_4_**	34	25	75	0.45	0.33
**2(QC_16_)_6_**	5	7	9	0.56	0.78
**CPC**	3	0.80	4	0.75	0.20
**BAB**	0.82	3	10	0.08	0.30

**Table 3:** List of compound abbreviations and corresponding SMILES notations used for in silico ADME profiling.

Compound	SMILES
**2(QO)_3_**	O=C(C1)C2CC[N+]1(CCC[N+]34CCC(C(C4)=O)CC3)CC2.[Br−].[Br−]
**2(QO)_4_**	O=C(C1)C2CC[N+]1(CCCC[N+]34CCC(C(C4)=O)CC3)CC2.[Br−].[Br−]
**2(QO)_6_**	O=C(C1)C2CC[N+]1(CCCCCC[N+]34CCC(C(C4)=O)CC3)CC2.[Br−].[Br−]
**2(QC_12_)_3_**	CCCCCCCCCCCNCC(C1)C2CC[N+]1(CCC[N+]34CCC(C(NCCCCCCCCCCCC)C4)CC3)CC2.[Br−].[Br−]
**2(QC_12_)_4_**	CCCCCCCCCCCNCC(C1)C2CC[N+]1(CCCC[N+]34CCC(C(NCCCCCCCCCCCC)C4)CC3)CC2.[Br−].[Br−]
**2(QC_12_)_6_**	CCCCCCCCCCCNCC(C1)C2CC[N+]1(CCCCCC[N+]34CCC(C(NCCCCCCCCCCCC)C4)CC3)CC2.[Br−].[Br−]
**2(QC_14_)_3_**	CCCCCCCCCCCCCCNC(C1)C2CC[N+]1(CCC[N+]34CCC(C(NCCCCCCCCCCCCCC)C4)CC3)CC2.[Br−].[Br−]
**2(QC_14_)_4_**	CCCCCCCCCCCCCCNC(C1)C2CC[N+]1(CCCC[N+]34CCC(C(NCCCCCCCCCCCCCC)C4)CC3)CC2.[Br−].[Br−]
**2(QC_14_)_6_**	CCCCCCCCCCCCCCNC(C1)C2CC[N+]1(CCCCCC[N+]34CCC(C(NCCCCCCCCCCCCCC)C4)CC3)CC2.[Br−].[Br−]
**2(QC_16_)_3_**	CCCCCCCCCCCCCCCCNC(C1)C2CC[N+]1(CCC[N+]34CCC(C(NCCCCCCCCCCCCCCCC)C4)CC3)CC2.[Br−].[Br−]
**2(QC_16_)_4_**	CCCCCCCCCCCCCCCCNC(C1)C2CC[N+]1(CCCC[N+]34CCC(C(NCCCCCCCCCCCCCCCC)C4)CC3)CC2.[Br−].[Br−]
**2(QC_16_)_6_**	CCCCCCCCCCCCCCCCNC(C1)C2CC[N+]1(CCCCCC[N+]34CCC(C(NCCCCCCCCCCCCCCCC)C4)CC3)CC2.[Br−].[Br−]
**OCT**	CCCCCCCCNC1=CC=[N+](CCCCCCCCCC[N+]2=CC=C(NCCCCCCCC)C=C2)C=C1.[Cl−].[Cl−]

## Data Availability

The original contributions presented in this study are included in the article. Further inquiries can be directed at the corresponding author.
